# *Alkalihalobacterium elongatum* gen. nov. sp. nov.: An Antibiotic-Producing Bacterium Isolated From Lonar Lake and Reclassification of the Genus *Alkalihalobacillus* Into Seven Novel Genera

**DOI:** 10.3389/fmicb.2021.722369

**Published:** 2021-10-11

**Authors:** Amaraja Joshi, Sonia Thite, Prachi Karodi, Neetha Joseph, Tushar Lodha

**Affiliations:** National Centre for Microbial Resource, National Centre for Cell Science, Pune, India

**Keywords:** alkaliphilic bacteria, *Alkalihalobacterium*, *Alkalihalobacillus*, taxogenomic, Lonar lake, *Shouchella*, *Halalkalibacterium*, *Halalkalibacter*

## Abstract

A Gram-stain positive, long, rod-shaped, motile, and spore-forming bacterium (MEB199^T^) was isolated from a sediment sample collected from Lonar Lake, India. The strain was oxidase and catalase positive. The strain grew optimally at pH 10, NaCl concentration of 3.5% at 37°C. The major fatty acids were *iso-*C_15:0_, *iso-*C_16:0_, *anteiso-*C_15:0_, and *iso-*C_17:0_. The peptidoglycan contained *meso*-diaminopimelic acid (*meso*-DAP). Phosphatidylethanolamine, diphosphatidylglycerol, and phosphatidylglycerol were the major polar lipids of MEB199^T^. Phylogenetic analysis based on 16S rRNA gene sequence showed that strain MEB199^T^ belonged to the family *Bacillaceae* and exhibited a distinctive position among the members of the genus *Alkalihalobacillus (Ahb.)*. Strain MEB199^T^ shared the highest 16S rRNA gene sequence similarity with *Alkalihalobacillus alkalinitrilicus* ANL-iso4^T^ (98.36%), whereas with type species *Ahb. alcalophilus* DSM 485^T^, it is 94.91%, indicating that strain MEB199^T^ is distinctly related to the genus *Alkalihalobacillus*. The G + C content of genomic DNA was 36.47 mol%. The digital DNA–DNA hybridization (dDDH) (23.6%) and average nucleotide identity (ANI) (81%) values between strain MEB199^T^ and *Ahb. alkalinitrilicus* ANL-iso4^T^ confirmed the novelty of this new species. The pairwise identity based on the 16S rRNA gene sequence between the species of genus *Alkalihalobacillus* ranges from 87.4 to 99.81% indicating the heterogeneity in the genus. The different phylogenetic analysis based on the genome showed that the members of the genus *Alkalihalobacillus* separated into eight distinct clades. The intra-clade average amino acid identity (AAI) and percentage of conserved proteins (POCP) range from 52 to 68% and 37 to 59%, respectively, which are interspersed on the intra-genera cutoff values; therefore, we reassess the taxonomy of genus *Alkalihalobacillus*. The phenotypic analysis also corroborated the differentiation between these clades. Based on the phylogenetic analysis, genomic indices, and phenotypic traits, we propose the reclassification of the genus *Alkalihalobacillus* into seven new genera for which the names *Alkalihalobacterium* gen. nov., *Halalkalibacterium* gen. nov., *Halalkalibacter* gen. nov., *Shouchella* gen. nov., *Pseudalkalibacillus* gen. nov., *Alkalicoccobacillus* gen. nov., and *Alkalihalophilus* gen. nov. are proposed and provide an emended description of *Alkalihalobacillus sensu stricto*. Also, we propose the *Ahb. okuhidensis* as a heterotypic synonym of *Alkalihalobacillus halodurans*. Based on the polyphasic taxonomic analysis, strain MEB199^T^ represents a novel species of newly proposed genus for which the name *Alkalihalobacterium elongatum* gen. nov. sp. nov. is proposed. The type strain is MEB199^T^ (= MCC 2982^T^, = JCM 33704^T^, = NBRC 114256^T^, = CGMCC 1.17254^T^).

## Introduction

The genus *Bacillus* is an extremely diverse group of bacteria within the phylum *Firmicutes* whose members currently exhibit great phylogenetic and phenotypic diversity. Numerous species that are part of this genus are unrelated to the type species as they do not share a common evolutionary history (La Duc et al., 2004). Recently, using phylogenomic approaches resolved the issue of the phylogenetic heterogeneity of the genus *Bacillus* by reclassifying existing species into novel genera, such as *Alkalihalobacillus (Ahb.)*, *Cytobacillus*, *Mesobacillus*, *Metabacillus*, *Neobacillus*, and *Peribacillus* (Patel and Gupta, 2020). Among all these genera, the genus *Alkalihalobacillus* consists of rod-shaped, endospore-forming, and Gram-stain-variable bacteria included in the family *Bacillaceae* with the type species *Alkalihalobacillus alcalophilus*. Based on phylogenomic studies, it was proposed that most of the members of the genus *Alkalihalobacillus* exclusively shared 10 CSIs found in the different proteins (Patel and Gupta, 2020). The genus *Alkalihalobacillus* contains 39 species.^1^ Most species of this genus are aerobic, but some members are facultative anaerobic and anaerobic. Species are found to be motile by peritrichous flagella, while a few members are non-motile. Members of genus *Alkalihalobacillus* were isolated from diverse environments including Soda lake soil/sediment, saltpan, hypersaline lake, mushroom compost, seawater, sea urchin, guts of larvae, feces, rhizosphere soil, non-saline forest soil, mud goldmine, mangrove sediment, mural paintings, etc., (Vedder, 1934; Nielsen et al., 1995; Li et al., 2002; Heyrman et al., 2003; Yumoto et al., 2003, 2005; Ivanova et al., 2004; Santini et al., 2004; Yoon et al., 2004; Nogi et al., 2005; Olivera et al., 2005; Vargas et al., 2005; Nowlan et al., 2006; Borchert et al., 2007; Ghosh et al., 2007; Sorokin et al., 2008; Aizawa et al., 2010; Denizci et al., 2010; Borsodi et al., 2011, 2017; Chen et al., 2011a, b; Madhaiyan et al., 2011; Zhang et al., 2011; Nedashkovskaya et al., 2012; Lei et al., 2014; Zhu et al., 2014; Reddy et al., 2015; Dou et al., 2016; Song et al., 2016; Singh et al., 2018; Liu et al., 2019; Gupta et al., 2020; Mo et al., 2020; Patel and Gupta, 2020; Shin et al., 2020). The majority of species from this genus are alkaliphilic and can grow in the pH range of 6–11 with optimum growth at pH 9–10. Some of the members are found to be obligately alkaliphilic in nature. The members are halotolerant or halophilic in nature as they grow in the presence of 1–5% w/v NaCl concentration. Members of this genus are mesophilic and grow at a temperature from 4 to 45°C with optimum growth at 25–37°C. Several species from this genus are of considerable industrial interest due to the production of enzymes such as cellulases, xylanases, proteases, and cyclodextrin glucanotransferase. *Ahb. rhizosphaerae* are diazotrophic, while *Ahb. clausii* exhibit probiotic activity (Nielsen et al., 1995; Madhaiyan et al., 2011).

While exploring the bacterial diversity of alkaline Lonar Lake, an antimicrobial compound producing alkaliphilic, moderately halophilic bacterial strain designated as MEB199^T^ was isolated from the sediment sample. Its taxonomic position was determined by employing a polyphasic taxonomic approach including whole genome-based analysis. During the assessment of the taxonomic status of the strain MEB199^T^, it was observed that the genome-based phylogenetic analysis and overall genome relatedness index (OGRI) indicated that the genus *Alkalihalobacillus* is composed of heterogeneous members, and its reclassification is required. Apart from having phylogenetic differences, members of the genus *Alkalihalobacillus* also differ in phenotypic characters such as morphology, growth requirement, polar lipids, and fatty acid composition. Based on phenotypic characteristics, phylogenetic analysis, and OGRI, we propose the reclassification of genus *Alkalihalobacillus* into seven new genera and provide an emended description of the genus *Alkalihalobacillus sensu stricto*. Similarly, the combined phenotypic and genotypic analysis indicate that the strain MEB199^T^ represents a novel species of the newly proposed genus *Alkalihalobacterium* gen. nov., for which the name *Alkalihalobacterium elongatum* gen. nov. sp. nov. is proposed. Based on digital DNA–DNA hybridization (dDDH) and ANI value, it was noticed that *Ahb. halodurans* DSM 497^T^ and *Ahb. okuhidensis* DSM 13666^T^ belong to the same species. Therefore, we propose *Ahb. okuhidensis* as a heterotypic synonym of *Ahb. halodurans*.

## Materials and Methods

### Sample Collection and Bacterial Isolation

Strain MEB199^T^ was isolated from a sediment sample collected from Lonar, an Indian soda lake situated at Buldhana District, Maharashtra, India, at a depth of 0.46 m (1.5 ft) on October 27, 2010. At the time of sampling, the pH of the sample was found to be 9.8 and temperature was 28°C. The sediment sample was serially diluted, spread on nutrient agar (pH 9.8; HiMedia, catalog no. M001), and incubated aerobically at 30°C. Bacterial colonies were observed after 2 days, which were purified after three successive transfers to a fresh medium. *Ahb. alkalinitrilicus* DSM 22532^T^ was procured from DSMZ German Collection of Microorganisms and Cell Cultures GmbH. *Desertibacillus haloalkaliphilus* KJ1-10-99^T^ was shared with us by Dr. Hitarth B. Bhatt, Saurashtra University, Rajkot, Gujarat, India, as a gratis. All strains were grown on nutrient agar (pH 9.8) and preserved as glycerol (20% v/v) stock, which was stored at −80°C and in liquid nitrogen.

### Phenotypic, Physiological, and Biochemical Characterization

The phenotypic characterization of MEB199^T^ and *Ahb. alkalinitrilicus* DSM 22532^T^ was carried out under the same laboratory conditions. Morphological characteristics were studied following the growth on nutrient agar (pH 9.8) media (HiMedia, catalog no. M001) plates incubated at 37°C for 48–72 h. Gram staining and spore staining were performed following standard procedures. Catalase and oxidase tests was carried as described earlier (Smibert and Krieg, 1994). Motility was checked by the hanging drop method. Hydrolysis of casein, starch, gelatin, nitrate, and nitrite were tested separately as reported previously (Smibert and Krieg, 1994). API 20E, API 20NE, API 50CH, API ZYM strips (bioMérieux, France), and BIOLOG GEN III plate were used to study the activities of constitutive enzymes, fermentation/oxidation profile, acid production, and substrate utilization as sole carbon and energy sources at 37°C for 48 h according to the instructions of the manufacturers. The temperature range for growth was determined on nutrient agar (pH 10) plates by incubating cultures at 4–45°C (4, 10, 20, 28, 37, and 45°C) for 72–96 h. Tolerance to various NaCl concentrations and pH were investigated using salt basal medium (SBM) as described earlier (Dimitriu et al., 2005) by measuring the optical densities (wavelength 600 nm) at 37°C up to 96 h. Tolerance to NaCl was tested using SBM with various NaCl concentrations (0–10%, w/v, at intervals of 0.5%). Growth was assessed in SBM adjusted to pH 7–11 at intervals of 0.5 pH unit by KH_2_PO_4_/K_2_HPO_4_ or Na_2_CO_3_/NaHCO_3_ buffer system. All parameters (temperature, NaCl concentration, and pH of the medium) were tested in triplicate.

### Chemotaxonomic Analyses

For cellular fatty acid analysis, strain MEB199^T^ and *Ahb. alkalinitrilicus* DSM 22532^T^ were grown on nutrient agar (pH 10) plates at 37°C for 16 h and collected at the same physiological age (at a logarithmic phase of growth). Cellular fatty acid methyl esters (FAMEs) were obtained from cells by saponification, methylation, and extraction following the protocol of MIDI. Cellular FAMEs were separated by gas chromatography (7890N, Agilent Technologies) and analyzed using the Sherlock Microbial Identification System (MIDI with database RTSBA6) according to the protocol described by the Sherlock Microbial Identification System. Cell wall samples were prepared from approximately 3 g of wet cells. Whole-cell hydrolyzates were prepared (6 M HCl, 100°C, 18 h) and examined by thin layer chromatography (TLC) on cellulose plates using n-butanol:water:acetic acid (50:25:25, v/v) as the solvent system.

Polar lipids were extracted from both the strains and analyzed. The cultures were harvested at a logarithmic phase, and the pellet was used for polar lipid extraction with methanol/chloroform/0.3% sodium chloride (2:1:0.8, by vol.) as described by Bligh and Dyer (1959) considering the modifications of Card (1973). Lipids were separated using silica gel TLC (Kieselgel 60 F254; Merck) by two-dimensional chromatography using chloroform:methanol:water (65:25:4 by vol.) in the first dimension and chloroform:acetic acid:methanol:water (40:7.5:6:2, by vol.) in the second dimension (Minnikin et al., 1984). The dried plates were subjected to spraying with 5% ethanolic phosphomolybdic acid for total lipids and further characterized by spraying with ninhydrin (specific for amino groups), molybdenum blue (specific for phosphates), Dragendorff (quaternary nitrogen), or α-naphthol (specific for sugars).

### Genomic DNA Isolation and 16S rRNA Gene Sequence Analysis

For DNA extraction, strain MEB199^T^ and *Desertibacillus haloalkaliphilus* KJ1-10-99^T^ were grown on nutrient agar (pH 10) medium and incubated at 37°C for 48–96 h. Genomic DNA was extracted as described earlier, and 16S rRNA genes were amplified using the universal primer set 27F (5′-AGAGTTTGATCMTGGCTCAG-3′) and 1488R (5′-CGGTTACCTTGTTACGACTTCACC-3′) (Brosius et al., 1978). Purified PCR products were sequenced on both strands on an ABI 3730 xl DNA analyzer using the Big Dye terminator kit (Applied Biosystems). The sequence was compared with the 16S rRNA gene sequence available at the EzBioCloud database (Yoon et al., 2017).

### Genome Sequencing and Annotations

The genomes of MEB199^T^ and *Desertibacillus haloalkaliphilus* KJ1-10-99^T^ were sequenced on the Illumina MiSeq (250 × 2 chemistry) platform. The reads were assembled using SPAdes (version 3.1.3.0), and the quality of the assembly was checked using QUAST (version 5.0.2). The obtained genome sequence was subsequently deposited in NCBI. The Gene prediction was performed using GeneMarkS and validated with the prokaryotic annotation pipeline of NCBI, PGAP. The Rapid Annotations using Subsystem Technology (RAST) server was used for the genome annotations.^2^ The GenBank accession numbers for the 16S rRNA gene sequence and draft genome sequence of strain MEB199^T^ are KX171019 and WMKZ00000000, respectively.

### Genome Sequences Used in This Study and Pathway Analysis

Type strains of 41 species of the genus *Alkalihalobacillus* (28 type strains), *Desertibacillus*, and *Anaerobacillus* and type species of the different genera of the family Bacilla, whose genomes were available in the public database, were considered in this study. Fifty genomes of non-type strains of genus *Alkalihalobacillus* were also included in this study. *Streptococcus gordonii* ATCC 10558^T^ and *Streptococcus agalactiae* ATCC 13813^T^ genomes were used to root (outgroup) the phylogenetic tree. The genome of strain MEB199^T^ and *Desertibacillus haloalkaliphilus* KJ1-10-99^T^ were sequenced under this study, and all the other genomes were downloaded from the PATRIC database (Supplementary Table 1). The functional annotations of each genome was carried out using EggNOG-mapper v2 (Cantalapiedra et al., 2021). The pathway was mapped using the KEGG Orthology (KO) Database^3^ for all the selected genomes. Heatmap to visualize the distribution of pathways across all the members of the genus *Alkalihalobacillus* was constructed using heatmapper.^4^

### Production of Secondary Metabolites

The antiSMASH bacterial version 6.0 was used to understand the secondary metabolite biosynthetic gene clusters in strain MEB199^T^ (Blin et al., 2021). To check the antibacterial activity of the compound produced by MEB199^T^, the aqueous extract of strain MEB199^T^ was tested for its activity against four multidrug-resistant (MDR, resistant to three or more antimicrobial classes) pathogens: *Acinetobacter baumannii* BAC01, *Escherichia coli* BAC03, *Staphylococcus aureus* MCC 2043^T^, and *Klebsiella pneumoniae* BAC02 using the agar overlay method. All the above pathogens used in the present study are clinical isolates resistant to more than six antibiotics. The strain MEB199^T^ was grown aerobically in nutrient broth (pH 10) medium under shaking conditions (150 rev min^–1^) for 96 h at 37°C. After incubation, the culture broth was centrifuged at 16,770 × *g* for 30 min. The supernatant was filtered through a filter of 0.22-μm pore size. The filtrate was concentrated 10-fold by lyophilization. The antimicrobial activity of the extract was carried out using a well diffusion method where 50 μl of the concentrated filtrate was added to wells (6-mm diameter) in Mueller–Hinton agar plates containing pathogenic indicator strains and incubated at 37°C for 96 h. The inhibition of growth was expressed as the diameter of the zone of inhibition around the well. All tests were carried out in triplicate.

### Phylogenetic Analysis

The 16S rRNA gene sequences of all the *Alkalihalobacillus* spp. and related members were retrieved from the NCBI database. Using the Up-to-date bacterial core gene (UBCG) tool, 92 core genes were extracted from all the genomes including two outgroups i.e., *Streptococcus gordonii* ATCC 10558^T^ and *Streptococcus agalactiae* ATCC 13813^T^ (Na et al., 2018). A concatenated sequence was used to construct the phylogenetic tree using MEGA7. The distance was calculated with Kimura two-parameter as a model of nucleotide substitution, in pairwise deletion procedure, Poisson model. The phylogenetic tree was constructed using the neighbor-joining (NJ), maximum-parsimony (MP), and maximum-likelihood (ML) methods with bootstrap analysis of 1,000 resamplings in the MEGA7 software package (Kumar et al., 2016). To assess the taxonomic position of the strain MEB199^T^, a codon tree based on 500 single-copy genes was reconstructed using amino acid and nucleotide sequences as described in Suresh et al. (2019). The Genome Taxonomy Database toolkit (GTDB-Tk) was used to construct the phylogenetic tree from the genome sequences (Chaumeil et al., 2019).

### Pan-Genome, Conserved Signature Indels, and Genomic Indices

The pan-genome of the species of the genus *Alkalihalobacillus* was analyzed by the Bacterial Pan Genome Analysis (BPGA) software (Chaudhari et al., 2016). To understand the interspecies variation and core genome, BPGA was used at its default parameters. Similarly, the pan-genome of the proposed genera was analyzed independently. Conserved signature indels (CSIs) were identified using protein sequences from the core proteins present in the members of the genus *Alkalihalobacillus* as described by Gupta (2014). Multiple sequence alignments were performed using Clustal Omega.^5^ The alignments were visually inspected for sequence gaps (insertion or deletion) of fixed lengths. Average nucleotide identity (ANI) analysis between the species of the genus *Alkalihalobacillus* was performed by using the ANI calculator.^6^ The dDDH was calculated using the genome-to-genome distance calculator using the HSP length, and formula 2 values were considered in this analysis (Goris et al., 2007). The percentage of conserved proteins (POCP) and average amino acid identity (AAI) for the genus level delineation were calculated. The POCP was calculated as described by Qin et al. (2014), and the AAI was computed using an online ANI/AAI-Matrix calculator.^7^

## Results and Discussion

### Isolation of Strain MEB199^T^ and Morphology

During the exploration of bacterial diversity of the alkaline saline Lonar Lake, a strain MEB199^T^ was isolated in nutrient agar (pH 10) from a sediment sample. Cells of strain MEB199^T^ showed 2- to 5-mm, cream-colored, flat, and dry colonies with irregular margins on nutrient agar (pH 10) medium after 48 h at 37°C. The cells of the strain MEB199^T^ was Gram stain positive, motile, long thick rods (6.4–16.5 × 0.6–2.4 μm), and spore forming. The strain was oxidase and catalase positive. The strain MEB199^T^ is alkaliphilic and halophilic, grew optimally at pH 10, at an NaCl concentration of 3.5%.

### Physiological Characterization of Strain MEB199^T^

The strain MEB199^T^ and *Ahb. alkalinitrilicus* DSM 22532^T^ were tested for utilization and assimilation of various carbon sources and enzyme activity against different substrates by API (bioMérieux, France) and BIOLOG GEN III plate. The differential morphological, physiological, and biochemical characteristics of strain MEB199^T^ and *Ahb. alkalinitrilicus* DSM 22532^T^ are given in Table 1. In the BIOLOG GEN III plate, MEB199^T^ was positive for various substrates like gentiobiose, D-melibiose, α-D-glucose, D-fructose, D-galactose, 3-methyl glucose, D-fucose, L-fucose, L-rhamnose, D-fructose-6-PO_4_, D-galacturonic acid, D-glucuronic acid, glucuronamid, and sodium butyrate. The strain is negative for acetoacetic acid and acetic acid, while its closest phylogenetic neighbor, *Ahb. alkalinitrilicus* DSM 22532^T^, was positive for those substrates. The strain MEB199^T^ showed a weak positive reaction for propionic acid, while *Ahb. alkalinitrilicus* DSM 22532^T^ showed negative activity for that substrate. In the API ZYM system, both the strains under study tested positive for the production of enzymes like leucine arylamidase, valine arylamidase, α-chymotrypsin, napthol AS-BI-phosphohydrolase, and α-glucosidase. *Ahb. alkalinitrilicus* DSM 22532^T^ could be able to produce enzymes like esterase (C4) and esterase lipase (C8), while MEB199^T^ could not. Acid phosphatases and ß-glucosidase were produced by MEB199^T^ and not found in *Ahb. alkalinitrilicus* DSM 22532^T^; this differentiated the novel strain from the type strain DSM 22532^T^. In the API 20E system, MEB199^T^ showed negative results for Voges Proskauer and sucrose fermentation, while *Ahb. alkalinitrilicus* showed positive results for both tests. Strain MEB199^T^ reduced nitrate to nitrite, hydrolyze esculin, and could assimilate mannitol and malate, while *Ahb. alkalinitrilicus* showed negative results for all those substrates but could assimilate maltose in the API 20 NE system. The physiological characteristics using BIOLOG GEN III and API analyses provided further support to investigate strain MEB199^T^ for its unique taxonomic position.

**TABLE 1:** Differential characteristics between strain MEB199^T^ and its phylogenetic neighbor.

Characteristics	1	2
Cell length (μm)	6.4–16.5	5–13
Cell width (μm)	0.6–2.4	0.6–2.2
Endospore	O	R
Motility	+	+
Temperature range (optimum),°C	20–45 (37)	20–45 (32)
pH range (optimum)	7–11 (10)	7–11 (9.5)
NaCl range (optimum),% (w/v)	0–10 (3.5)	0–7 (1)
**Oxidation/reduction of**		
Acetoacetic acid	−	+
Acetic acid	−	+
Propionic acid	+(w)	−
Voges Proskauer	−	+(w)
Sucrose fermentation	−	+
**Assimilation of**		
Mannitol	+	−
Malate	+	−
Maltose	−	+
Reduction of nitrate	+	−
Hydrolysis of esculin	+	−
**API–Zym tests**		
Esterase (C4)	−	+
Esterase lipase (C8)	−	+
Acid phosphatase	+	−
ß-glucosidase	+	−
**Polar lipids**		
APL	−	+
PL1	+	−
PL2	−	+
PL3	−	+
PL5	−	+

*1, Strain MEB199^T^; 2, Alkalihalobacillus alkalinitrilicus DSM 22532^T^; +, Positive; −, negative; w, weakly positive; O, oval subterminal; R, round subterminal; APL, unidentified amino phospholipid; PL, unidentified phospholipid lipid. All data were obtained from this study unless otherwise indicated.*

### Chemotaxonomic Analyses of Strain MEB199^T^

Chemotaxonomic characteristics of strain MEB199^T^ were consistent with those of members of the family *Bacillaceae*. The cellular fatty acid composition of strain MEB199^T^ showed a spectrum of 12 fatty acids with a pronounced dominance of *iso-*C_15:0_ (27.4%), *iso-*C_16:0_ (13.4%), *anteiso-*C_15:0_ (11.8%), and *iso-*C_17:0_ (10.5%). The fatty acids were dominated by branched and monounsaturated fatty acids. *Ahb. alkalinitrilicus* DSM 22532^T^ showed *anteiso-*C_15:0_, *iso-*C_15:0_, -*iso-*C_16:0_, and *iso-*C_14:0_ as primary fatty acids with *anteiso-*C_15:0_ as the dominant one, while MEB199^T^ has *iso-*C_15:0_ the dominant fatty acid. Apart from the major fatty acids in strain MEB199^T^, other qualitative and quantitative differences were observed in the reference strains with respect to other minor fatty acids (Table 2). The polar lipid profile of strain MEB199^T^ was found to contain diphosphatidylglycerol, phosphatidylglycerol, phosphatidylethanolamine, and an unidentified phospholipid lipid (Supplementary Figure 1). Polar lipids of strain MEB199^T^ differed from strain DSM 22532^T^ by the absence of unidentified aminolipid (APL) and unidentified phospholipid (PL2, PL3, PL4, and PL5). The peptidoglycan of strain MEB199^T^ contained *meso*-diaminopimelic acid (*meso*-DAP) as the cell wall diamino acid. The peptidoglycan diamino acid of the cell wall of strains MEB199^T^ and *Ahb. alkalinitrilicus* DSM 22532^T^ were similar to those found in members of the genus *Alkalihalobacillus*.

**TABLE 2:** Comparison of the fatty acid composition of strain MEB199^T^ and its phylogenetic neighbor.

Fatty acids	1	2
*iso-*C_14:0_	2.9	**10.9**
*iso-*C_15:0_	**27.4**	**21.8**
*anteiso-*C_15:0_	**11.8**	**30.3**
C_16:1_ω7c alcohol	1.8	3.2
*iso-*C_16:0_	**13.4**	**17**
C_16:0_	5.8	3
*iso-*C_17:1_ω10c	4.2	1.3
*iso-*C_17:1_ω5c	3.8	ND
*iso-*C_17:0_	**10.5**	2.7
*anteiso-*C_17:0_	5.86	5.13
18:0	1	ND
Summed Feature 3^*a*^	4.5	1

*1, Strain MEB199^T^; 2, Alkalihalobacillus alkalinitrilicus DSM 22532^T^; ND, not detected.*

*Results are presented as a percentage of the total fatty acids. Fatty acids amounting to 10% or more of the total fatty acids are in bold. Values of less than 1% for all strains are not shown.*

*^a^Summed features are groups of two or three fatty acids that could not be separated by GC with the MIDI system. Summed feature 3 comprised C_16:1_ω6c and/or C_16:1_ω7c.*

### Genomic Features of Strain MEB199^T^

Paired-end sequencing resulted in about 1,983,695 quality-filtered reads with an average read length of 224.33 bp. Assembly of reads resulted in 61 contigs with a total sequence length of 4.81 Mbp. The sequencing coverage was approximately 232X. The DNA G + C content was determined from the genome sequence, which is 36.7%. Annotation of the genome consisted of 4,926 coding sequences. The protein-coding genes of MEB199^T^ have an average length of 776 bases, ranging from 56 to 7,274 bases. Strain MEB199^T^ harbor only one copy of the 16S rRNA gene (1,551 bp). Out of 4,926 open reading frames (ORFs) identified, 3,156 (64.06%) were functionally annotated, with 1,770 (35.93%) being hypothetical genes. The 16S rRNA gene sequence extracted from whole-genome was compared with that determined by PCR and Sanger sequencing (KX171019). Both sequences were found identical.

### 16S rRNA Sequence and Analysis

The complete (1,551 bp) 16S rRNA gene sequence of strain MEB199^T^ was used for sequence and phylogenetic analysis. Based on EzTaxon-e search analysis, the closest phylogenetic neighbor of strain MEB199^T^ is *Ahb. alkalinitrilicus* DSM 22532^T^, with which it shared 98.36% sequence similarity, followed by *Desertibacillus haloalkaliphilus* KJ1-10-99^T^ (97.10%), *Anaerobacillus alkaliphilus* B16-10^T^ (96.72%), and *Anaerobacillus isosaccharinicus* NB2006^T^ (96.59%). It showed a similarity of <96% with other species. However, the pairwise sequence identity level between strain MEB199^T^ and *Ahb. alcalophilus* DSM485^T^, the type species of the genus *Alkalihalobacillus*, was 94.91%, which indicates that strain MEB199^T^ might not be a member of the genus *Alkalihalobacillus*.

### 16S rRNA Phylogeny

**FIGURE 1:**
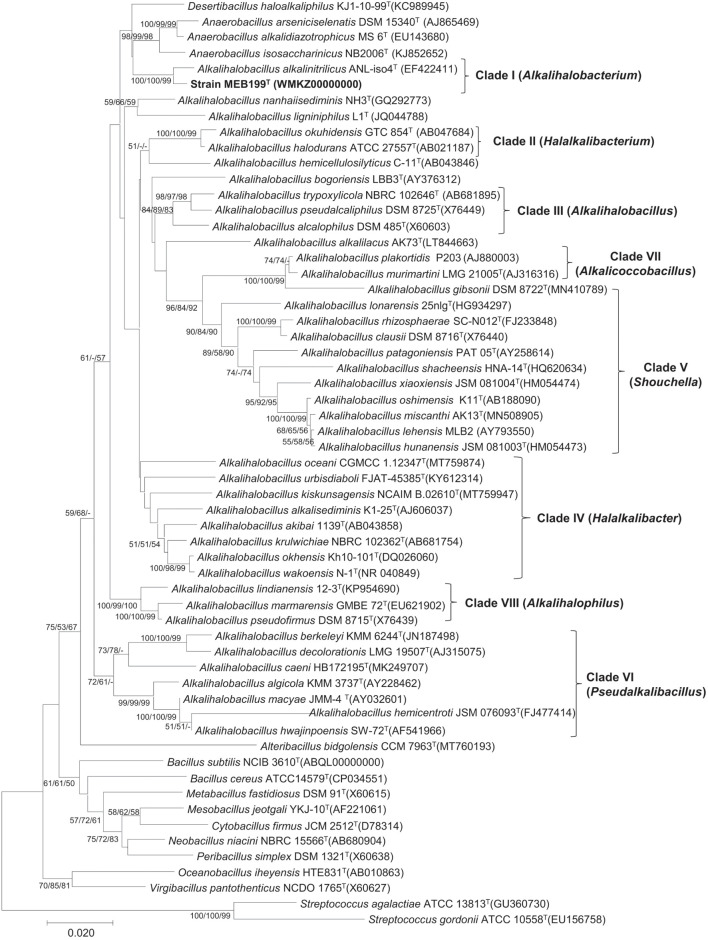
Phylogenetic tree based on 16S rRNA gene sequence showing the phylogenetic relationship between the members of the genus *Alkalihalobacillus*. The tree was constructed using MEGA7 and *Streptococcus gordonii* ATCC 10558^T^, and *Streptococcus agalactiae* ATCC 13813^T^ was used as an outgroup. The 16S rRNA gene bank accession number is shown in parentheses. The bootstrap percentage refers to minimum-evolution (ME)/neighbor-joining (NJ)/maximum-likelihood (ML) analysis. The bootstrap values only above 50 for each node are indicated. Scale bar indicates the number of substitutions per site.

The phylogenetic tree based on the 16S rRNA gene placed strain MEB199^T^ and a closely related *Ahb. alkalinitrilicus* DSM 22532^T^ in a separate clade (Figure 1). The phylogenetic trees constructed using MP and ML methods revealed a similar tree topology with the common node with *Ahb. alkalinitrilicus* DSM 22532^T^, which confirmed a close similarity between these two strains. The genus *Alkalihalobacillus* was separated into eight different clades, which were referred to as Clade I, Clade II, Clade III, Clade IV, Clade V, Clade VI, Clade VII, and Clade VIII in the subsequent discussion (Figure 1). It is interesting to point out that the 16S rRNA gene sequence similarities between species of the genus *Alkalihalobacillus* ranged from 87.40 to 99.81% (Supplementary Table 2). The pairwise distance of 16S rRNA gene sequence identity value of <95 indicates affiliation with different genera (Rosselló-Móra and Amann, 2015). This wide range (87.40–99.81%) of 16S rRNA gene sequence similarity indicates heterogeneity in the genus *Alkalihalobacillus* and signposts the need to reassess the taxonomy of the genus. The pairwise sequence identity among the *Alkalihalobacillus* species showed that 16S rRNA gene sequences have limited power, and to better resolve the taxonomic affiliation in the members of the genus *Alkalihalobacillus*, other approaches available in the genomic era have to be investigated.

### Whole-Genome Phylogeny

**FIGURE 2:**
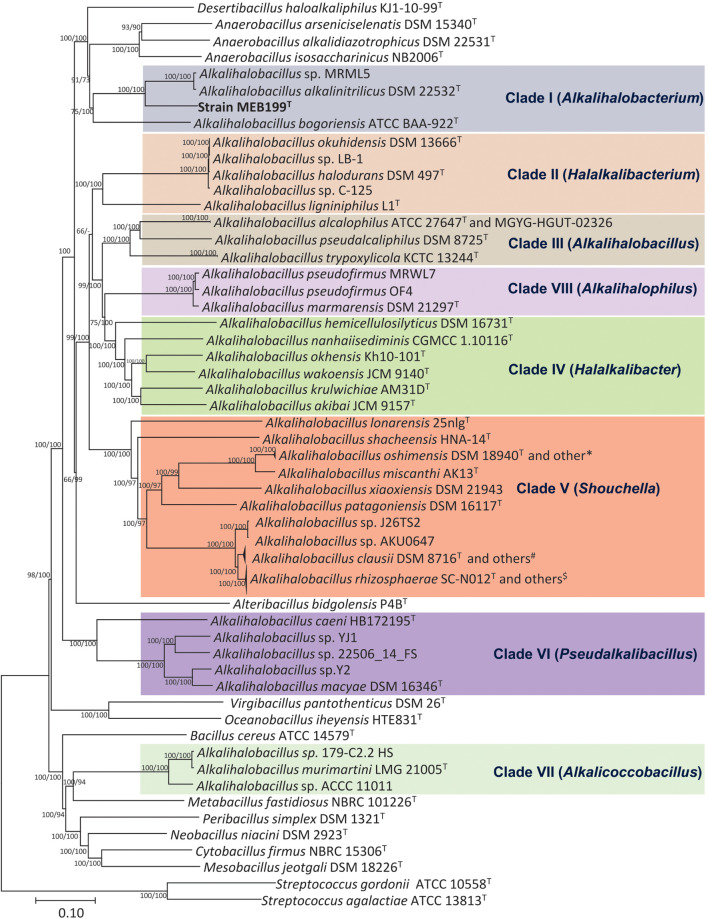
Phylogenetic tree constructed using the 92 bacterial core gene sequences showing the relationships of the members of genus *Alkalihalobacillus* and nearest genera. The 92 gene sequences were extracted using Up-to-date bacterial core gene (UBCG) tool, which is a widely used resource for delineating the phylogeny of bacteria, and the phylogenetic tree was constructed using MEGA7 with NJ and ME algorithms. Bar, 0.1 nucleotide substitution per position. *Strains DSM 19099, G25-134, G1, DSM 19153; ^#^Strains 7520-2, 7540-2, 7547-G, 179-F 5B1 HS, 7535-K, 7541, 7538, 7523-2, 088AE, BC112, 7522; ^$^Strains 7894-1, UBBC-07, J32TS2, 7529, KSM-K16, 7540-1, 7539, 7537-T, J1TS1, UBBC-08/C, UBBC-08/T, UBBC-08/R, GMN, B637/NM, B619/R, B603/Nb, CSI08, ENTPro, UBBC-08/S, B106, 7543.

Phylogenomic analysis based on the core genome made up of 92 genes and codon tree of the recently described genera by Patel and Gupta (2020) as well as the remaining species in the genus *Alkalihalobacillus* formed well-supported eight clades (Figure 2 and Supplementary Figure 2). The phylogenomic tree based on 120 ubiquitous single-copy proteins was also constructed using GTDB-Tk, which also separates the genus *Alkalihalobacillus* into eight clades (Supplementary Figure 3). Strain MEB199^T^ clustered together with *Ahb. alkalinitrilicus* DSM 22532^T^ and *Ahb. bogoriensis* ATCC BAA-922^T^ by forming a separate clade distinguishable from genus *Alkalihalobacillus*. Based on the phylogenomic analysis, Clade I, Clade II, Clade III, Clade IV, Clade V, Clade VI, Clade VII, and Clade VIII were observed similar to the 16S rRNA gene sequence-based analysis. *Ahb. murimartini* LMG 21005^T^ was present in Clade VII in the 16S rRNA gene-based tree within the members of genus *Alkalihalobacillus*, but in phylogenomic analysis, it was completely outgrouped from the genus *Alkalihalobacillus* (Figure 2).

### Analysis of Core and Pan-Genome of the Genus *Alkalihalobacillus*

**FIGURE 3:**
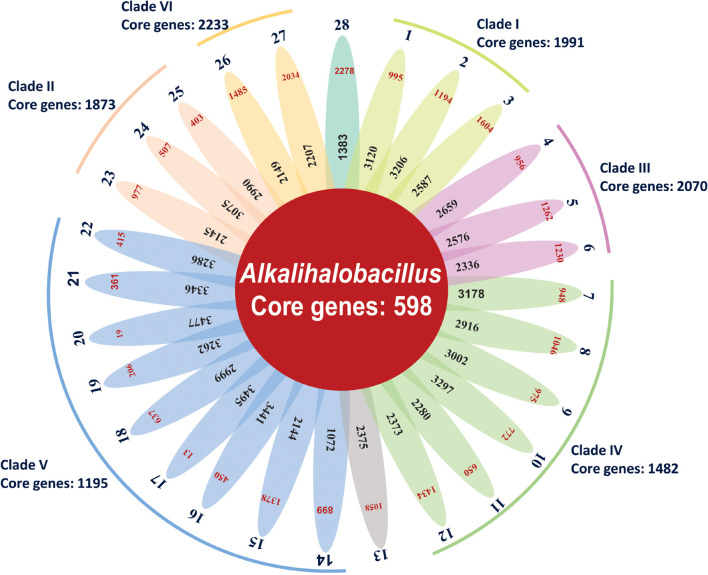
Flower-pot diagram representing core, accessory, and unique genomes of type strains of genus *Alkalihalobacillus* (1, Strain MEB199^T^; 2, *Ahb. alkalinitrilicus* DSM 22532^T^; 3, *Ahb. bogoriensis* ATCC BAA-922^T^; 4, *Ahb. alcalophilus* ATCC 27647^T^; 5, *Ahb. pseudalcaliphilus* DSM 8725^T^; 6, *Ahb. trypoxylicola* KCTC 13244^T^; 7, *Ahb. akibai* JCM 9157^T^; 8, *Ahb. krulwichiae* AM31D^T^; 9, *Ahb. wakoensis* JCM 9140^T^; 10, *Ahb. okhensis* Kh10-101^T^; 11, *Ahb. nanhaiisediminis* CGMCC 1.10116^T^; 12, *Ahb. hemicellulosilyticus* DSM 16731^T^; 13, *Ahb. marmarensis* DSM 21297^T^; 14, *Ahb. lonarensis* 25nlg^T^; 15, *Ahb. shacheensis* HNA-14^T^; 16, *Ahb. clausii* DSM 8716^T^; 17, *Ahb. oshimensis* DSM 18940^T^; 18, *Ahb. patagoniensis* DSM 16117^T^; 19, *Ahb. lehensis* DSM 19099; 20, *Ahb. plakortidis* DSM 19153; 21, *Ahb. rhizosphaerae* SC-N012^T^; 22, *Ahb. miscanthi* AK13^T^; 23, *Ahb. ligniniphilus* L1^T^; 24, *Ahb. okuhidensis* DSM 13666^T^; 25, *Ahb. halodurans* DSM 497^T^; 26, *Ahb. macyae* DSM 16346^T^; 27, *Ahb. caeni* HB172195^T^ and 28, *Ahb. murimartini* LMG 21005^T^).

Bacterial pan-genome analysis was carried out between the type strains of the genus *Alkalihalobacillus*. In the 28 analyzed species of the genus *Alkalihalobacillus*, 598 genes were identified as core genes indicating that members of the genus *Alkalihalobacillus* share a very small number of core genes. The core genes encode for the shikimate pathway, isoprenoid biosynthesis (non-mevalonate pathway), thiamine biosynthesis, tetrahydrofolate biosynthesis, pantothenate biosynthesis, lysine biosynthesis, riboflavin biosynthesis, inosine monophosphate biosynthesis, uridine monophosphate biosynthesis, F-type ATPase, heme biosynthesis, pyrimidine deoxyribonucleotide biosynthesis, citrate cycle (TCA cycle, Krebs cycle), lysine biosynthesis, coenzyme A biosynthesis, glycolysis (Embden–Meyerhof pathway), pantothenate biosynthesis, gluconeogenesis, glycolysis, pyruvate oxidation, adenine ribonucleotide biosynthesis, NAD biosynthesis, guanine ribonucleotide biosynthesis, UDP-*N*-acetyl-D-glucosamine biosynthesis, and dicarboxylate–hydroxybutyrate cycle. The core genome analysis was also carried out for the individual clade formed in the phylogenomic tree. Clade I shares 1,991 as core genes with 3,024 genes as accessory genomes. Clade II shares 1,873 core genes with 3,128 accessory genes (Figure 3 and Table 3). Clade III shares 2,070 core genes and 1,942 as accessory genes. Clade IV shares 1,482 as core genes, and Clade V shares 1,195 core genes. Clade VI consists of only two members of the genus *Alkalihalobacillus*, which shares 2,233 core genes between them (Figure 3 and Table 3). In the clade-wise pan-genome analysis, there was an increase in the number of core genes, which showed the divergence in inter-clade genomes.

**TABLE 3:** The ranges of intra-clade genomic indices.

Name of clade	ANI^*a*^ (%)	dDDH^*a*^ (%)	POCP^*a*^ (%)	AAI^*a*^ (%)	G + C content (%)	Pairwise identity^*b*^ (%)	Core genes
Clade I	76–81	20–24	53–72	64–81	36.7–36.83	94.25–98.86	1991
Clade II	77–99	20–94	57–83	67–98	40.8–43.2	94.81–99.43	1873
Clade III	78	20–20	60–68	67–73	35.7–37.7	97.68–98.76	2070
Clade IV	77–88	19–28	52–73	63–80	37.0–39.8	95.13–99.7	1482
Clade V	75–95	18–63	48–89	61–97	39.6–47.1	91.01–99.81	1195
Clade VI	76	19	62	64	39.8–40.9	90.65–98.48	2233

*ANI, average nucleotide identity; dDDH, digital DNA–DNA hybridization; POCP, percentage of conserved proteins; AAI, average amino acid identity.*

*^a^For Clade V the values between Alkalihalobacillus oshimensis DSM 18940^T^, Alkalihalobacillus plakortidis DSM 19153, and Alkalihalobacillus lehensis DSM 19099 were not considered as they are synonyms.*

*^b^The pairwise identity between the 16S rRNA gene sequence.*

### Functional Analysis and Significance of Genus *Alkalihalobacillus*

The members of the genus *Alkalihalobacillus* are an industrially important group of bacteria, which have been isolated from diverse ecological niches (Jones et al., 1998; Grant, 2003). They are likely to play an important but yet unexplored role in the functional stability and maintenance of the ecosystem. Several species from this genus are of considerable industrial interest due to their production of enzymes such as cellulases, proteases for inclusion in laundry detergents, xylanases for use in the pulp paper industry, and cyclodextrin glucanotransferase for manufacture of cyclodextrin from starch (Horikoshi, 2006). The genus *Alkalihalobacillus* is attracting interest because its members have a great biotechnological potential for producing compatible solutes or hydrolytic enzymes (Horikoshi, 1999; Margesin and Schinner, 2001; Arahal and Ventosa, 2002; Krulwich et al., 2007). Some of these bacterial species are believed to have industrial potential as a source of alkali-stable enzymes (Gessesse and Gashe, 1997). *Ahb. patagoniensis*, *Ahb. lehensis*, and *Ahb. marmarensis* are producers of alkaline proteases, while *Ahb. lonarensis* and *Ahb. oshimensis* could produce various protease, lipase, and xylanase enzymes. Obligately alkaliphilic species, *Ahb. krulwichiae*, can degrade aromatic compounds in alkaline conditions, while *Ahb. ligniniphilus*, a halotolerant alkaliphilic bacterium, is used to degrade lignin (Zhu et al., 2017). *Ahb. rhizosphaerae*, which is diazotrophic and can fix atmospheric nitrogen to ammonia and other species such as *Alkalihalobacillus clausii*, exhibits probiotic activity due to the production of antimicrobial compounds (Nielsen et al., 1995; Madhaiyan et al., 2011). The strain MEB199^T^ also showed an antimicrobial compound-producing activity.

**FIGURE 4:**
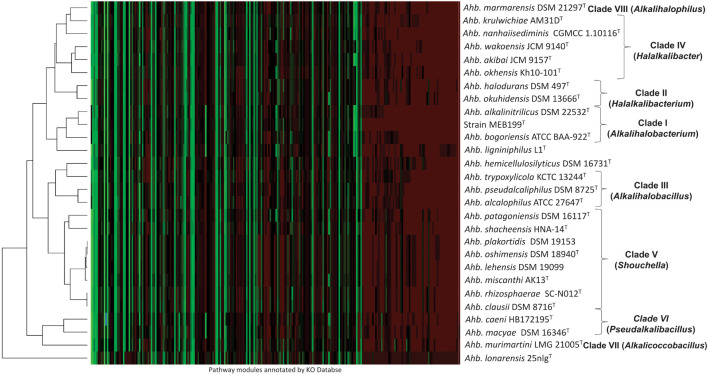
Heatmap showing functional potential of all the members of the genus *Alkalihalobacillus*. Functional annotations were performed using EggNOG, and pathways were reconstructed using the KEGG Orthology (KO) Database server, and heatmap was generated by the Heatmapper using average linkage clustering method and Spearman rank correlation distance measurement method. The color variations depict the relative abundance of genes in the pathways wherein red denotes the maximally abundant pathways, and green represents the least abundant pathways.

The heatmap clustering based on the distributions of metabolic pathways in the genomes is shown in Figure 4. The clusters formed in the heatmap corroborate the clades in phylogenomic analysis except *Ahb. ligniniphilus* L1^T^, *Ahb. hemicellulosilyticus* DSM 16731^T^, and *Ahb. lonarensis* 25nlg^T^. The genome mining showed that the 11 subunits of NADH:quinone oxidoreductase (*nuoA*, *nuoB*, *nuoC*, *nuoD*, *nuoH*, *nuoI*, *nuoJ*, *nuoK*, *nuoL*, *nuoM*, and *nuoN*) was exclusively present in the members of Clade I, Clade VI, and *Alkalihalobacillus ligniniphilus* L1^T^ and absent in all the other members of genus *Alkalihalobacillus*. The genetic capability to synthesize the isoprenoid compounds and terpenoid was screened in the genomes. All the members of the genus *Alkalihalobacillus* harbors methylerythritol phosphate (MEP) i.e., non-mevalonate pathway for the synthesis of isoprenoid precursors isopentenyl pyrophosphate (IPP). IPP is the precursor for the various isoprenoid molecules playing diverse roles in different processes in the bacterial cell. Menaquinones, a lipid-soluble quinone, are isoprenoid compounds that participate in the electron transport chain. All the members of the genus *Alkalihalobacillus* has the menaquinone (MK) biosynthesis pathway, but an alternative futalosine pathway to convert chorismate to 1,4-dihydroxy-6-naphthoate requires four enzymes encoded by mqnABCD, which are absent in Clade VI (*Ahb. macyae* DSM 16346^T^ and *Ahb. caeni* HB172195^T^) and *Ahb. murimartini* LMG 21005^T^. The classical MK pathway is exclusively present in all aerobic and facultatively anaerobic bacteria in contrast to the futalosine pathway, which is present in aerobic and anaerobic bacteria. All the Clade I, Clade VI, and VII members have sporulenol synthase gene, which gives the genetic capability to cyclize tetraprenyl beta-curcumene into sporulenol (C35 terpenes), a pentacyclic sesquarterpene (Sato et al., 2011). Sporulenol, produced during the sporulation, is present in the spores and increases the resistance to reactive oxygen species (Kontnik et al., 2008).

### Antimicrobial Activity

The bacterial strain MEB199^T^ produced antibacterial metabolites against MDR pathogens (*Acinetobacter baumannii* BAC01, *Escherichia coli* BAC03, *Staphylococcus aureus* MCC 2043^T^, and *Klebsiella pneumoniae* BAC02) and was shown by the zone of inhibition (Supplementary Figure 4). The antiSMASH analysis showed that the strain MEB199^T^ has 12 secondary metabolite biosynthetic gene clusters for the synthesis of siderophore, terpene (carotenoids and thailanstatin A), lasso peptide (paeninodin), lanthipeptide-class-I (streptin), beta lactone (fengycin), type III polyketide synthases (T3PKS) (7-deoxypactamycin), ribosomally synthesized and post-translationally modified peptides [linear azole(in)e-containing peptides LAP], and ectoine, whereas *Ahb. alkalinitrilicus* DSM 22532^T^ has 8 biosynthetic gene clusters for the synthesis of siderophore, terpene (carotenoid), LAP (RiPP-like), lasso peptide (paeninodin), beta-lactone (fengycin), T3PKS (7-deoxypactamycin), and ectoine but the aqueous extract of strain DSM22532^T^ did not show antimicrobial activity against MDR strains, which separate it from the strain MEB199^T^. All the genomes of genus *Alkalihalobacillus* were screened for the presence of secondary metabolite biosynthetic gene clusters. All members of the genus *Alkalihalobacillus* have multiple biosynthetic gene clusters ranging from 3 to 12 (Supplementary Table 3). All the studied members of genus *Alkalihalobacillus* have T3PKS biosynthetic gene cluster coding for 7-deoxypactamycin, a new member of the pactamycin group except *Ahb. bogoriensis* ATCC BAA-922^T^. *Ahb. murimartini* LMG 21005^T^ has 12 biosynthetic gene clusters out of which 8 are unique and not present in other members of genus *Alkalihalobacillus*. There is heterogeneity in the distribution of biosynthetic gene clusters in the genus *Alkalihalobacillus*. There is no clade-wise pattern observed in the antiSMASH analysis as the secondary metabolite production is strain-specific character.

### Use of Average Nucleotide Identity and Digital DNA–DNA Hybridization for Species Delineation

**FIGURE 5:**
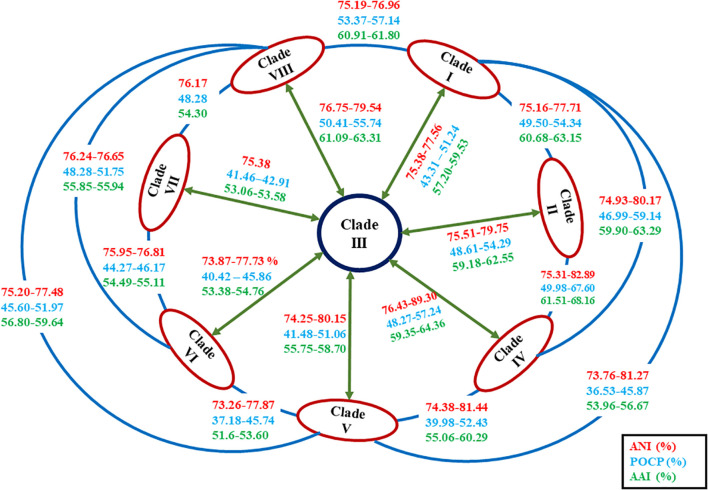
Diagrammatic representation of the average nucleotide identity (ANI), average amino acid identity (AAI), and percentage of conserved proteins (POCP) values in between the members of each clade of the genus *Alkalihalobacillus*.

The ANI value between MEB199^T^ and *Ahb. alkalinitrilicus* strain DSM 22532^T^ was 81%. The *in silico* dDDH was carried out using the genome-to-genome distance calculator^8^ between the strain MEB199^T^ and *Ahb. alkalinitrilicus* DSM 22532^T^. The dDDH analysis using HSP length showed 24% relatedness between MEB199^T^ and *Ahb. alkalinitrilicus* DSM 22532^T^. From ANI and dDDH analysis, it can be inferred that MEB199^T^ is a novel species. On the other hand, the ANI level between strain MEB199^T^ and the type species of the genus *Alkalihalobacillus* was determined as 76% indicating its distant affiliation to the genus *Alkalihalobacillus*. The results of the calculations of the ANI and dDDH among the studied genomes are given in Supplementary Table 4. The results of ANI and dDDH calculations showed that the genomes grouped into the same clusters observed by the analyses of core genes and phylogenomics. Intra-clade ANI and dDDH values ranges from 75 to 95% and 18 to 63%, respectively (Table 3). All the ANI values between the species of genus *Alkalihalobacillus* was >96 except ANI between *Ahb. okuhidensis* DSM 13666^T^ and *Ahb. halodurans* DSM 497^T^, which is 99%. This explicitly indicates that defined species of the genus are delineating from each other except *Ahb. okuhidensis* DSM 13666^T^ and *Ahb. halodurans* DSM 497^T^, which failed to delineate from each other at the species level and belong to the same species. Each of the eight clusters showed that inter-clade ANI values ranged between 73.26 and 89.0% (Figure 5 and Table 3). These values are relatively similar to those reported by Qin et al. that found 68–82% interspecies ANI values among the different genera (Qin et al., 2014). The dDDH values for the species of the genus ranged from 17.8 to 63.2%, which are <70% except between *Ahb. okuhidensis* DSM 13666^T^ and *Ahb. halodurans* DSM 497^T^. The dDDH between *Ahb. okuhidensis* DSM 13666^T^ and *Ahb. halodurans* DSM 497^T^ is 94%, which indicates that these two belong to the same species of the genus *Alkalihalobacillus*.

### Use of Percentage of Conserved Proteins and Average Amino Acid Identity Values for Genera Delineation

In order to reassess the taxonomic position of the species belonging to the genus *Alkalihalobacillus* and newly isolated strain MEB199^T^, genome-based comparisons for conserved protein-coding genes were performed by calculating AAI levels between strain MEB199^T^ and its close neighbors using the AAI matrix calculator. AAI values between strain MEB199^T^ and its closest neighbors *Ahb. alkalinitrilicus* DSM 22532^T^and *Ahb. bogoriensis* ATCC BAA-922^T^ were calculated as 81 and 64%, respectively, while AAI values between MEB199^T^ and other type strains of the genus *Alkalihalobacillus* were lower than 60% (Supplementary Table 5). POCP values between strain MEB199^T^ and its closest neighbors *Ahb. alkalinitrilicus* DSM 22532^T^ and *Ahb. bogoriensis* ATCC BAA-922^T^ were calculated as 72 and 57%, respectively, while POCP values were <50% for the type species of the genus *Alkalihalobacillus* as well as between the newly described genera in the family *Bacillaceae* (Figure 5 and Supplementary Table 5). Consequently, strain MEB199^T^, together with its close phylogenetic neighbors, i.e., *Ahb. alkalinitrilicus* DSM 22532^T^ and *Ahb. bogoriensis* ATCC BAA-922^T^, are considered to represent a novel genus within the family *Bacillaceae*.

To confirm whether the clades observed in the phylogenomic tree might represent different genera, the genomic indices POCP and AAI were also calculated with the species of genus *Alkalihalobacillus* (Supplementary Table 5). Considering recent work on genus delineation based on mean protein sequence similarity of all protein-coding genes, members of the family *Bacillaceae* can be distinguished by 65–70% AAI value at the genus level (Aliyu et al., 2016). The inter-clade AAI values ranged from 52 to 68% indicating that the genus *Alkalihalobacillus* is divergent and polyphyletic (Figure 5 and Supplementary Table 5). Although Qin et al. (2014) proposed <50% POCP to delineate the genera, POCP values between different newly proposed *Bacillus* genera ranged from 34.8 to 69.8% (Aliyu et al., 2016; Patel and Gupta, 2020). The inter-clade POCP values ranged from 37 to 68% (Figure 5 and Supplementary Table 5). The AAI and POCP values also indicate that the members of the genus *Alkalihalobacillus* are divergent, and there is a need for reclassification of the genus *Alkalihalobacillus*. Each clade in phylogenomic analysis represents a novel genus. A detailed survey of the phenotypic characters was carried out to determine if the description of new taxa at the genus level is possible, or such clades were only clusters or genomovars within the genus *Alkalihalobacillus*. Because the genomic analysis of 52 *Arcobacter cryaerophilus* strains indicate four different genomospecies, but the phenotypic study failed to delineate the species, therefore, they were considered genomovars of the same species (Pérez-Cataluña et al., 2018).

### Conserved Signature Indels Specific for Different Monophyletic Clades of *Alkalihalobacillus* Species

Phylogenetic analysis (16S rRNA gene-based and genome-based) indicated the existence of polyphyletic clades of genus *Alkalihalobacillus*. The conserved signature insertions and deletions (CSIs) in the proteins are the rare genetic changes that are exclusively shared by evolutionary linked organisms. CSIs are useful molecular signatures for evolutionarily and taxonomic studies. Therefore, the CSIs specific for the novel clades of members of the *Alkalihalobacillus* was studied. Patel and Gupta have reported that six CSIs are the signature of the genus *Alkalihalobacillus* (Patel and Gupta, 2020), whereas four CSI signatures in protein transcription–repair coupling factor, tRNA uridine-5-carboxymethylaminomethyl (34) synthesis enzyme (mnmG), 50S ribosomal protein L11 methyltransferase, and homoserine kinase were exclusively shared by all the members of the genus *Alkalihalobacillus* except *Ahb. alkalinitrilicus* and *Ahb. bogoriensis*. These results separate Clade I from the rest of the members of the genus *Alkalihalobacillus*. In the present study, CSIs that are distinctive characteristics of other clades were identified. The species of Clade II harbor the CSI of a two-amino acid insertion in the protein translocase subunit (secD) protein, which is absent in other members of the genus *Alkalihalobacillus* (Supplementary Figure 5). Members of Clade III consist of the CSI of a two-amino acid insertion in the UDP-*N*-acetylmuramoyl-L-alanyl-D-glutamate-2, 6-diaminopimelate ligase (murE) protein, which is absent in other members of the genus *Alkalihalobacillus* (Supplementary Figure 6). Two CSIs exclusively present in the members of Clade IV and absent in other species of the genus *Alkalihalobacillus* were identified. Insertion of two amino acids in DNA mismatch repair (mutL) protein and one amino acid deletion in ATP-dependent protease ATPase subunit (hslU) protein were observed in the species of Clade IV (Supplementary Figures 7, 8). Similarly, two CSIs were exclusively present in the species of Clade V, which are five-amino acid insertion in the protein translocase subunit (secY) protein and one-amino acid insertion in ATP-dependent protease ATPase subunit (hslU) protein (Supplementary Figures 9, 10). These CSIs in Clades I, II, III, IV, and V also showed the genetic distinctness of these clades formed in phylogenetic analysis and support the reclassification. We were not able to identify any CSIs for Clades VI, VII, and VIII.

### Phenotypes to Support Reclassifications

Phylogenomic analysis as well as genomic indices, indicated that *Alkalihalobacillus* species are too divergent to be placed into the same genus. To support this further, phenotypic and chemotaxonomic markers were also surveyed to further understand its taxonomic position. The comparison of phenotypic characters between the clades and nearest genera is given in Table 4. The phenotypic characters are also able to distinguish these clades, as the members of Clade I are isolated from a soda lake, alkaliphilic, long thick rods, Gram-stain positive, G + C content range from 35.1 to 37.5%, and strictly aerobic in nature, whereas members of Clade II are facultative anaerobes, Gram-stain variable, G + C content range from 40.76 to 43.9 mol%, and motile (Table 4; Nielsen et al., 1995; Li et al., 2002; Vargas et al., 2005; Sorokin et al., 2008; Zhu et al., 2014). Members of Clade IV are alkaliphilic, quinone (MK-5, MK-6, or MK-7), the optimum temperature is 37°C, oxidase negative, and contain glycolipid, which makes it unique from the rest of the members of the genus *Alkalihalobacillus* (Yumoto et al., 2003; Nogi et al., 2005; Nowlan et al., 2006; Borsodi et al., 2011, 2017; Zhang et al., 2011; Song et al., 2016; Liu et al., 2019; Gupta et al., 2020). Clade V is composed of mesophilic and neutrophilic organisms that differentiate them from the rest of the *Alkalihalobacillus* species (Nielsen et al., 1995; Olivera et al., 2005; Yumoto et al., 2005; Ghosh et al., 2007; Chen et al., 2011a; Madhaiyan et al., 2011; Lei et al., 2014; Reddy et al., 2015; Singh et al., 2018; Gupta et al., 2020; Shin et al., 2020). Members of Clade VI can grow both aerobically and anaerobically and alkalitolerant with optimum growth at pH 7, whereas other members are alkaliphilic in nature (Heyrman et al., 2003; Ivanova et al., 2004; Santini et al., 2004; Yoon et al., 2004; Chen et al., 2011b; Nedashkovskaya et al., 2012; Mo et al., 2020). *Ahb. murimartini* LMG 21005^T^ is a neutrophilic, coccoidal-shaped bacterium that separates it from the rest of the members of the genus *Alkalihalobacillus* (Borchert et al., 2007). These phenotypic differences also indicate that there is a need for reclassification of the genus *Alkalihalobacillus*.

**TABLE 4:** Comparison of seven newly proposed genera with genus *Alkalihalobacillus sensu stricto* and members of closely related genera of the family *Bacillaceae*.

Characteristic	1	2	3	4	5	6	7	8	9	10	11	12	13	14	15	16
Gram nature	+	±	+	±	+	±	±	+	+	+	+	±	±	±	±	±
Cell shape	Rod	Rod	Rod	Rod	Rod	Rod	Coccoid rod	Rod	Rod with round ends	Rod	Rod	Rod	Rod	Rod	Rod	Rod
Motility	±	+	−	±	±	±	+	+	−	±	±	+	±	±	±	+
Aerobic/anaerobic	Strictly aerobic	Facultative anaerobic	Aerobic	Aerobic or anaerobic	Aerobic or facultative anaerobic	Aerobic/anaerobic/facultative anaerobic	Aerobic	Aerobic	Aerobic	Strictly anaerobic/aerotolerant	Strictly aerobic	Aerobic or facultative anaerobic	Aerobic or facultative anaerobic	Aerobic	Aerobic or facultative anaerobic	Aerobic or facultative anaerobic
pH range (optimum)	8–11 (9–10)	6–11 (9–10)	8–10 (9)	6.5–12.5 (9–10)	6–11 (8–9)^a^	5–10 (7–8)^b^	7–11 (8)	8–12 (9)	7–10 (9)	8.5–10.7 (9.6–9.7)	6.5–10 (7–8)	6–10 (7–8)	7–11 (7–8)	6–10 (7–8)^c^	5–10 (7–8)	5.5–9 (5.5–6.5)
NaCl range (optimum),% w/v	0–11.6 (2.5)	0–12 (2.0)	5–10 (2)	0–12 (3–7)	0–22 (5–10)	0–11 (3–6)	0–10 (4–7)	0–12 (4)	0–20 (7.5)	0–110 (20–30)	0.5–24.0 (5–10)	0–10 (0–4)	0–13 (0–5)	0–25.0 (0–15.0)	0–15 (0–7.5)	0–12 (5)
Temp range (optimum,°C)	10–45 (32–37)	15–60 (30–40)	10–40 (30)	10–45 (37)	4–50 (25–35)	4–50 (25–40)	5–40 (25–37)	10–45 (37)	20–55 (37)	10–40 (25–37)	20–50 (35–40)	10–45 (25–37)	10–45 (25–37)	4–45 (25–37)	15–50 (25–37)	3–45 (25–37)
Fatty acids	Iso-C_15:0_, anteiso-C_15:0_, iso-C_14:0_, iso-C_16:0_, iso-C_17:0_	Iso-C_14:0_, anteiso-C_15:0_	Iso-C_15:0_, anteiso-C_15:0_, anteiso-C_17:0_, iso-C_17:0_	Anteiso-C_15:0_, iso-C_15:0_, C_16:0_, anteiso-C_17:0_, C_14:0_, C_16:1_ω7c alcohol, C_16:0_	Iso-C15:0, anteiso-C15:0, iso-C17:0 and anteiso-C17:0, iso-C14: 0, C16:0, C12:0	Anteiso-C_15:0_, anteiso-C_17:0_, C_16:1_ω7c alcohol	Anteiso-C_15:0_, anteiso-C_17:0_	Anteiso-C_15:0_, iso C_15:0_	Anteiso-C_15:0_, anteiso-C_17:0_, C_16:0_, iso-C_15:0_	C_14:0_, iso-C_14:0_, C_16:0_, C_16:1_ω7c, C_16:1_ω11c, anteiso-C_15:0_, iso-C_15:0_, C_16:1_ω7c/iso-C_15:0_2OH, C_18:1_ω7c	Iso-C_15:0_, anteiso-C_15:0_	Iso-C_15:0_, anteiso-C_15:0_, iso-C_14:0_	Iso-C_15:0_, anteiso-C_15:0_	Iso-C_15:0_, anteiso-C_15:0_	Iso-C_15:0_, anteiso-C_15:0_	Anteiso-C_15:0_, iso-C_14:0_, iso-C_15:0_
Quinones	MK-7	MK-7	MK-7	MK-5,6,7	MK-7	MK-7	ND	MK-8	MK-7	MK-7	MK-7, 8	MK-7	MK-7	MK-7	MK-7	MK-7
Polar lipids	DPG, PE, PG, APL, PL	PG, DPG, PE	DPG, PE, PG, PL	PE, PG, DPG, APL, GL	DPG,PG, PE GL	PG, DPG, PE	ND	PG, DPG, PE	DPG, PG	PG, PE, DPG	PG, APL, PL	DPG, PE, PG	DPG, PE, PG	DPG, PE, PG	DPG, PE, PG	DPG, PE, PG
Genome size	4.81–5.51	3.86–4.21	4.21–4.49	4.3–4.74	2.58–4.65	4.2–4.7	4.17	3.86–4.02	4.43	3.95–4.95	3.32–4.7	4.5–5.6	4.2–5.7	4.1–5.3	3.2–6.1	4.3–5.6
Mole G + C, mole%	35.1–37.5	40.7–43.9	36.2–39	34.4–41.6	39.7–54	37–40.9	39⋅8	39.0–42.7	48.9	40.0	37.1–38.9	38.1–40.8	33.7–45.4	35.1–44.4	37.3–43.7	37.4–43.0

*Taxa: 1, Clade I (Ahb. alkalinitrilicus, Ahb. bogoriensis and MEB199T; data from this study, Vargas et al., 2005; Sorokin et al., 2008; Patel and Gupta, 2020); 2, Clade II (Ahb. halodurans, Ahb. Ligniniphilus, and Ahb. okuhidensis; data from Nielsen et al., 1995; Li et al., 2002; Zhu et al., 2014; Patel and Gupta, 2020); 3, Clade III (Ahb. alcalophilus, Ahb. Pseudalcaliphilus, and Ahb. trypoxylicola; data from Vedder, 1934; Nielsen et al., 1995; Aizawa et al., 2010; Patel and Gupta, 2020); 4, Clade IV (Ahb. krulwichiae, Ahb. hemicellulosilyticus, Ahb. nanhaiisediminis, Ahb. wakoensis, Ahb. okhensis, Ahb. akibai, Ahb. oceani, Ahb. kiskunsagensis, Ahb. alkalisediminis, and Ahb. urbisdiaboli; data from Yumoto et al., 2003; Nogi et al., 2005; Nowlan et al., 2006; Borsodi et al., 2011, 2017; Zhang et al., 2011; Song et al., 2016; Liu et al., 2019; Gupta et al., 2020); 5, Clade V (Ahb. clausii, Ahb. rhizosphaerae, Ahb. patagoniensis, Ahb. miscanthi, Ahb. plakortidis, Ahb. oshimensis, Ahb. lehensis, Ahb. shacheensis, Ahb. lonarensis, Ahb. alkalilacus, Ahb. gibsonii, Ahb. Xiaoxiensis, and Ahb. hunanensis; data from Nielsen et al., 1995; Olivera et al., 2005; Yumoto et al., 2005; Ghosh et al., 2007; Chen et al., 2011a; Madhaiyan et al., 2011; Lei et al., 2014; Reddy et al., 2015; Singh et al., 2018; Gupta et al., 2020; Shin et al., 2020); 6, Clade VI (Ahb. caeni, Ahb. decolorationis, Ahb. macyae, Ahb. hemicentroti, Ahb. hwajinpoensis, Ahb. Algicola, and Ahb. berkeleyi; data from Heyrman et al., 2003; Ivanova et al., 2004; Santini et al., 2004; Yoon et al., 2004; Chen et al., 2011b; Nedashkovskaya et al., 2012; Mo et al., 2020); 7, Clade VII (Ahb. murimartini; data from Borchert et al., 2007; Patel and Gupta, 2020); 8, Clade VIII (Ahb. pseudofirmus, Ahb. marmarensis, and Ahb. lindianensis; data from Nielsen et al., 1995; Denizci et al., 2010; Dou et al., 2016; Patel and Gupta, 2020); 9, Desertibacillus (Bhatt et al., 2017). 10, Anaerobacillus (Zavarzina et al., 2009); 11, Alteribacillus (Azmatunnisa et al., 2016); 12, Cytobacillus (Patel and Gupta, 2020); 13, Mesobacillus (Patel and Gupta, 2020); 14, Metabacillus (Patel and Gupta, 2020); 15, Neobacillus (Patel and Gupta, 2020); 16, Peribacillus (Patel and Gupta, 2020).*

*PE, phosphatidylethanolamine; PG, phosphatidylglycerol, GL, glycolipid; DPG, diphosphatidylglycerol; APL, amino phospholipid; PL, unknown phospholipid; +, positive/present; −, negative/absent; MK, menaquinone; ND, no data available.*

*^a^Ahb. miscanthii grows optimally pH at 7.*

*^b^Ahb. algicola and Ahb. berkeleyi grow optimally at pH 9.*

*^c^Metabacillus lacus grows at pH 7–12 and optimally at pH 9.*

## Conclusion

Based on phenotypic, genomic, phylogenetic, and chemotaxonomic characteristics, we propose the reclassification of genus *Alkalihalobacillus* into seven new genera with an emended description of the genus *Alkalihalobacillus sensu stricto*. We propose members of Clade I to be classified into a new genus for which we propose the name *Alkalihalobacterium* gen. nov. Though genomic indices showed that *Ahb. bogoriensis* is distinctly related to members of Clade I, we prefer to transfer *Ahb. bogoriensis* into *Alkalihalobacterium* gen. nov. for practical reasons to separate new genera until more strains related to this taxon become available. Therefore, *Alkalihalobacterium alkalinitrilicus* comb. nov. is proposed as type species of the newly proposed genus *Alkalihalobacterium* gen. nov. Clade II showed that the AAI, POCP, and phenotypic traits are showing clear delineation from other members. Hence, we propose that the members of Clade II be classified into the genus *Halalkalibacterium* gen. nov. with *Halalkalibacterium halodurans* as type species of the genus. Moreover, as *Ahb. okuhidensis* DSM 13666^T^ and *Ahb. halodurans* DSM 497^T^ have high genomic indices (ANI, dDDH), we concluded that they are not different species. Based on the phenotypic differences, we propose *Ahb. okuhidensis* as a heterotypic synonym of *Ahb. halodurans*. Clade III harbors *Ahb. alcalophilus* ATCC 27647^T^, which is the type species of the genus *Alkalihalobacillus*; therefore, the members of Clade III are included in the genus *Alkalihalobacillus sensu stricto*. Clade IV encompasses *Ahb. hemicellulosilyticus*, *Ahb. nanhaiisediminis*, *Ahb. wakoensis*, *Ahb. okhensis*, *Ahb. krulwichiae*, *Ahb. akibai*, *Ahb. oceani, Ahb. kiskunsagensis*, *Ahb. alkalisediminis*, and *Ahb. urbisdiaboli* for we propose *Halalkalibacter* gen. nov. For Clade V, we propose *Shouchella* gen. nov. to accommodate *Ahb. rhizosphaerae*, *Ahb. clausii*, *Ahb. patagoniensis*, *Ahb. miscanthi*, *Ahb. plakortidis*, *Ahb. oshimensis*, *Ahb. lehensis*, *Ahb. shacheensis*, *Ahb. lonarensis*, *Ahb. alkalilacus*, *Ahb. gibsonii*, *Ahb. xiaoxiensis*, and *Ahb. hunanensis* species. Genomic analysis also corroborated a previous finding that *Ahb. plakortidis* DSM 19153 and *Ahb. lehensis* DSM 19099 are heterotypic synonyms of *Ahb. oshimensis* DSM 18940^T^. For Clade VI, we propose *Pseudalkalibacillus* gen. nov. to accommodate *Ahb. caeni*, *Ahb. macyae*, *Ahb. hemicentroti*, *Ahb. hwajinpoensis*, *Ahb. algicola*, *Ahb. berkeleyi*, and *Ahb. decolorationis* species. To accommodate *Ahb. murimartini* LMG 21005^T^, we propose the new genus *Alkalicocobacillus* gen. nov. The type strains, *Ahb. lindianensis* 12-3^T^, *Ahb. marmarensis* GMBE 72^T^, and *Ahb. pseudofirmus* DSM 8715^T^, formed a distinct clade in 16S rRNA gene sequence-based phylogeny. In genome-based phylogeny, *Ahb. marmarensis* DSM 21297^T^ gets outgrouped to the Clade IV. AAI between *Ahb. marmarensis* DSM 21297^T^ and other members of the Clade IV ranges from 63 to 68%, which indicates that it belongs to a distinct genus. Therefore, we propose members of Clade VIII as a novel genus for which name *Alkalihalophilus* gen. nov. is proposed. The description of the newly proposed genera is given below, and a description of all names and new combinations is given in Table 5.

**TABLE 5:** Description of the new combinations in the newly proposed genus.

New name combination and etymology	Basonym	Description	Type strain
**Description of the new combinations in the genus *Alkalihalobacterium***			
*Alkalihalobacterium alkalinitrilicum* comb. nov. (Type species of this genus) (al.ka.li.ni.tri’li.cum. N.L. n. *alkali* (from Arabic *al* the; *qaliy* soda ash) alkali; N.L. masc. adj. *nitrilicus* pertaining to nitriles; N.L. neut. adj. *alkalinitrilicum* alkaliphile utilizing nitriles)	*Bacillus alkalinitrilicus* (Sorokin et al., 2008) Patel and Gupta, 2020	The description of this taxon is as given by Sorokin et al. (2008)	ANL-iso4^T^ (= DSM 22532 = NCCB 100120 = UNIQEM U240)
*Alkalihalobacterium bogoriense* comb. nov. (bo.gor.i.en’se. N.L. neut. adj. *bogoriense* pertaining to Lake Bogoria, a soda lake in Kenya)	*Alkalihalobacillus bogoriensis* (Vargas et al., 2005) Patel and Gupta, 2020	The description of this taxon is as given by Vargas et al. (2005)	LBB3^T^ (= ATCC BAA-922 = MG 22234)
**Description of the new combinations in the genus *Halalkalibacterium***			
*Halalkalibacterium halodurans* comb. nov. (Type species of this genus) (ha.lo.du’rans. Gr. n. *hals halos* salt; L. pres. part. *durans* enduring; N.L. part. adj. *halodurans* salt-enduring)	*Alkalihalobacillus halodurans* (ex Boyer et al., 1973) (Nielsen et al., 1995) Patel and Gupta, 2020	The description of this taxon is as given by Nielsen et al. (1995)	PN-80^T^ (= ATCC 27557 = CIP 105296 = DSM 497 = LMG 7121 = NRRL B-3881)
*Halalkalibacterium ligniniphilum* comb. nov. (lig.ni.ni’phi.lum. N.L. neut. n. *ligninum* lignin; N.L. masc. adj. *philus* (from Gr. masc. adj. *philos*) friend, loving; N.L. neut. adj. *ligniniphilum* lignin-loving, isolated as a lignin degrader with lignin as a single carbon source)	*Alkalihalobacillus ligniniphilus* (Zhu et al., 2014) Patel and Gupta, 2020	The description of this taxon is as given by Zhu et al. (2014)	L1^T^ (= DSM 26145 = JCM 18543)
**Description of the new combinations in the genus *Halalkalibacter***			
*Halalkalibacter krulwichiae* comb. nov. (Type species of this genus) (krul.wich’i.ae. N.L. fem. gen. n. *krulwichiae* of Krulwich; named after American microbiologist Terry A. Krulwich who made fundamental contributions to the study of alkaliphilic bacteria)	*Alkalihalobacillus krulwichiae* (Yumoto et al., 2003) Patel and Gupta, 2020	The description of this taxon is as given by Yumoto et al. (2003)	AM31D^T^ (= DSM 18225 = IAM 15000 = JCM 11691 = NBRC 102362 = NCIMB 13904)
*Halalkalibacter oceani* comb. nov. (o.ce.a’ni. L. gen. masc. n. *oceani*, of an ocean, referring to its optimal growth under marine conditions)	*Alkalihalobacillus oceani* (Song et al., 2016) Gupta et al., 2020	The description of this taxon is as given by Song et al. (2016)	SW109^T^ (= CGMCC 1.12347 = DSM 100579)
*Halalkalibacter hemicellulosilyticus* comb. nov. (he.mi.cel.lu.lo.si.ly’ti.cus N.L. neut. n. *haemicellulosum*, hemicellulose; Gr. masc. adj. *lytikos*, able to loosen, able to dissolve; N.L. masc. adj. *hemicellulosilyticus*, hemicellulose-dissolving)	*Alkalihalobacillus hemicellulosilyticus* corrig. (Nogi et al., 2005) Patel and Gupta, 2020	The description of this taxon is as given by Nogi et al. (2005)	C-11^T^ (= DSM 16731 = JCM 9152)
*Halalkalibacter nanhaiisediminis* comb. nov. (nan.hai.i.se.di’mi.nis. N.L. neut. n. *nanhaium* Nan Hai, the Chinese name for the South China Sea; L. neut. n. *sedimen -inis* a sediment; N.L. gen. n. *nanhaiisediminis* of a sediment from the South China Sea)	*Alkalihalobacillus nanhaiisediminis* (Zhang et al., 2011) Patel and Gupta, 2020	The description of this taxon is as given by Zhang et al. (2011)	NH3^T^ (= CGMCC 1.10116 = DSM 27953 = JCM 16507)
*Halalkalibacter wakoensis* comb. nov. (wa.ko.en’sis. N.L. masc. adj. *wakoensis* of Wako, a city in Japan)	*Alkalihalobacillus wakoensis* (Nogi et al., 2005) Patel and Gupta, 2020	The description of this taxon is as given by Nogi et al. (2005)	N-1^T^ (= DSM 2521 = JCM 9140)
*Halalkalibacter okhensis* comb. nov. (ok.hen’sis. N.L. masc. adj. *okhensis* pertaining to Port Okha, a port of the Dwarka region in India, where the type strain was isolated)	*Alkalihalobacillus okhensis* (Nowlan et al., 2006) Patel and Gupta, 2020	The description of this taxon is as given by Nowlan et al. (2006)	GMBE 72^T^ (= DSM 21297 = JCM 15719)
*Halalkalibacter akibai* comb. nov. (a.ki.ba’i. N.L. gen. n. *akibai* of Akiba, named after the Japanese microbiologist Teruhiko Akiba, who made fundamental contributions to the study of alkaliphilic bacteria)	*Alkalihalobacillus akibai* (Nogi et al., 2005) Patel and Gupta, 2020	The description of this taxon is as given by Nogi et al. (2005)	1139^T^ (= ATCC 43226 = DSM 21942 = JCM 9157)
*Halalkalibacter kiskunsagensis* comb. nov. (kis.kun.sag.en’sis N.L. masc. adj. *kiskunsagensis*, referring to the name of Kiskunság National Park in Hungary, the location of the sampling site)	*Alkalihalobacillus kiskunsagensis* (Borsodi et al., 2017) Gupta et al., 2020	The description of this taxon is as given by Borsodi et al. (2017)	B16-24^T^ (= DSM 29791 = NCAIM B.02610)
*Halalkalibacter urbisdiaboli* comb. nov. (ur.bis.di.a’bo.li. L. fem. n. *urbs*, city; L. masc. n. *diabolus*, devil; N.L. gen. n. *urbisdiaboli*, of Devil City)	*Alkalihalobacillus urbisdiaboli* (Liu et al., 2019) Gupta et al., 2020	The description of this taxon is as given by Liu et al. (2019)	FJAT-45385^T^ (= CCTCC AB 2016263 = DSM 104651)
*Halalkalibacter alkalisediminis* comb. nov. (al.ka.li.se.di’mi.nis. N.L. n. *alkali* (from Arabic *al* the; *qaliy* soda ash) alkali; L. gen. n. *sediminis* of sediment; N.L. gen. n. *alkalisediminis* of alkaline sediment)	*Alkalihalobacillus alkalisediminis* (Borsodi et al., 2011) Patel and Gupta, 2020	The description of this taxon is as given by Borsodi et al. (2011)	K1-25^T^ (= DSM 21670 = NCAIM B.02301)
**Description of the new combinations in the genus *Shouchella***			
*Shouchella clausii* comb. nov. (Type species of this genus) (clau’si.i. N.L. gen. n. *clausii* of Claus, named after Dieter Claus, the German bacteriologist who made fundamental contributions to the taxonomy of *Bacillus*)	*Alkalihalobacillus clausii* (Nielsen et al., 1995) Patel and Gupta, 2020	The description of this taxon is as given by Nielsen et al. (1995)	PN-23^T^ (= ATCC 700160 = CCUG 47262 = CIP 104718 = DSM 8716 = LMG 17945 = NCIB 10309 = NCIMB 10309)
*Shouchella xiaoxiensis* comb. nov. (xi.a.o.xi.en’sis. N.L. fem. adj. *xiaoxiensis* pertaining to Xiaoxi National Natural Reserve, China, the source of the sample from which the type strain was isolated)	*Alkalihalobacillus xiaoxiensis* (Chen et al., 2011b) Patel and Gupta, 2020	The description of this taxon is as given by Chen et al. (2011b)	DSM 21943^T^ (= JSM 081004 = CCTCC AA 208057)
*Shouchella alkalilacus* comb. nov. (al.ka.li.la’cus. N.L. n. *alkali* (from Arabic *al* the; *qaliy* soda ash) alkali; L. gen. masc. n. *lacus*, a lake; N.L. gen. masc. n. *alkalilacus*, of an alkaline lake)	*Alkalihalobacillus alkalilacus* (Singh et al., 2018) Gupta et al., 2020	The description of this taxon is as given by Singh et al. (2018)	AK73^T^ (= JCM 32184 = KCTC 33880 = MTCC 12637)
*Shouchella rhizosphaerae* comb. nov. (rhi.zo.sphae’rae. N.L. gen. n. *rhizosphaerae* of the rhizosphere)	*Alkalihalobacillus rhizosphaerae* (Madhaiyan et al., 2011) Patel and Gupta, 2020	The description of this taxon is as given by Madhaiyan et al. (2011)	SC-N012^T^ (= DSM 21911 = NCCB 100267)
*Shouchella patagoniensis* comb. nov. (pa.ta.go.ni.en’sis. N.L. fem. adj. *patagoniensis* pertaining to Patagonia, in Argentina, where the type strain was isolated)	*Alkalihalobacillus patagoniensis* (Olivera et al., 2005) Patel and Gupta, 2020	The description of this taxon is as given by Olivera et al. (2005)	PAT 5^T^ (= ATCC BAA-965 = DSM 16117)
*Shouchella miscanthi* comb. nov. (misc.an’thi. N.L. gen. *masc*. n. *miscanthi*, of *Miscanthus sacchariflorus*, where the type strain was isolated)	*Alkalihalobacillus miscanthi* (Shin et al., 2020) Gupta et al., 2020	The description of this taxon is as given by Shin et al. (2020)	AK13^T^ (= DSM 109981 = KACC 21401)
*Shouchella plakortidis* comb. nov. (pla.kor’ti.dis. N.L. gen. n. *plakortidis* of *Plakortis*, a genus of sponges)	*Alkalihalobacillus plakortidis* (Borchert et al., 2007) Patel and Gupta, 2020	The description of this taxon is as given by Borchert et al. (2007)	P203^T^ (= DSM 19153 = NCIMB 14288)
*Shouchella oshimensis* comb. nov. (o.shi.men’sis. N.L. fem. adj. *oshimensis* from Oshima, the region where the micro-organism was isolated)	*Alkalihalobacillus oshimensis* (Yumoto et al., 2005) Patel and Gupta, 2020	The description of this taxon is as given by Yumoto et al. (2005)	K11 ^T^ (= DSM 18940 = JCM 12663 = NCIMB 14023)
*Shouchella lehensis* comb. nov. (le.hen’sis. N.L. fem. adj. *lehensis* pertaining to Leh, in India, where the type strain was isolated)	*Alkalihalobacillus lehensis* (Ghosh et al., 2007) Patel and Gupta, 2020	The description of this taxon is as given by Ghosh et al. (2007)	MTCC 7633^T^ (= MLB2 = JCM 13820 = LMG 24751 = DSM 19099)
*Shouchella shacheensis* comb. nov. (sha.che.en’sis. N.L. fem. adj. *shacheensis* pertaining to Shache County, Xinjiang Province, China, the source of the sample from which the type strain was isolated)	*Alkalihalobacillus shacheensis* (Lei et al., 2014) Patel and Gupta, 2020	The description of this taxon is as given by Lei et al. (2014)	HNA-14^T^ (= DSM 26902 = KCTC 33145)
*Shouchella lonarensis* comb. nov. (lo.nar.en’sis. N.L. fem. adj. *lonarensis* of or belonging to Lonar lake, India, from where the type strain was isolated)	*Alkalihalobacillus lonarensis* (Reddy et al., 2015) Patel and Gupta, 2020	The description of this taxon is as given by Reddy et al., 2015	LMG 27974^T^ (= CGMCC 1.12817 = KCTC 33413 = 25 nlg)
*Shouchella hunanensis* comb. nov. (hu.nan.en’sis. N.L. fem. adj. *hunanensis*, pertaining to Hunan Province, PR China, the source of the sample from which the type strain was isolated)	*Alkalihalobacillus hunanensis* Patel and Gupta, 2020	The description of this taxon is as given by Patel and Gupta (2020)	JSM 081003^T^ (= DSM 23008 = KCTC 13711)
*Shouchella gibsoni* comb. nov. (gib.so’ni.i. N.L. gen. masc. n. *gibsonii*, of Gibson, named after the British bacteriologist Thomas Gibson)	*Alkalihalobacillus gibsonii* (Nielsen et al., 1995) Gupta et al., 2020	The description of this taxon is as given by Nielsen et al. (1995)	PN-109^T^ (= ATCC 700164 = CIP 104720 = DSM 8722 = LMG 17949)
**Description of the new combinations in the genus *Pseudalkalibacillus***			
*Pseudalkalibacillus decolorationis* comb. nov. (Type species of this genus) (de.co.lo.ra.ti.o’nis. L. gen. n. *decolorationis* of discoloration)	*Alkalihalobacillus decolorationis* (Heyrman et al., 2003) Patel and Gupta, 2020	The description of this taxon is as given by Heyrman et al. (2003)	LMG 19507^T^ (= DSM 14890)
*Pseudalkalibacillus macyae* comb. nov. (ma’cy.ae. N.L. fem. n. *macyae* of Macy, named after the late Professor Joan M. Macy, Chair of Microbiology, La Trobe University, in tribute to her research in the area of environmental microbiology)	*Alkalihalobacillus macyae* (Santini et al., 2004) Patel and Gupta, 2020	The description of this taxon is as given by Santini et al. (2004)	JMM-4^T^ (= DSM 16346 = JCM 12340)
*Pseudalkalibacillus caeni* comb. nov. (cae’ni. L. gen. neut. n. *caeni*, of mud, referring to the source of isolation)	*Alkalihalobacillus caeni* (Mo et al., 2020) Gupta et al., 2020	The description of this taxon is as given by Mo et al. (2020)	HB172195^T^ (= CGMCC 1.16730 = JCM 33411)
*Pseudalkalibacillus hemicentroti* comb. nov. (he.mi.cen.tro’ti. N.L. gen. n. haemicentroti of Haemicentrotus (*Haemicentrotus pulcherrimus*, a sea urchin), the source of isolation of the organism)	*Alkalihalobacillus haemicentroti* (Chen et al., 2011a) Patel and Gupta, 2020	The description of this taxon is as given by Chen et al. (2011a)	JSM 076093^T^ (= DSM 23007 = KCTC 13710)
*Pseudalkalibacillus hwajinpoensis* comb. nov. (hwa.jin.po.en’sis. N.L. masc. adj. *hwajinpoensis* of Hwajinpo, a beach of the East Sea in Korea, where the type strain was isolated)	*Alkalihalobacillus hwajinpoensis* (Yoon et al., 2004) Patel and Gupta, 2020	The description of this taxon is as given by Yoon et al. (2004)	SW-72^T^ (= DSM 16206 = JCM 11807 = KCCM 41641 = LMG 24749)
*Pseudalkalibacillus algicola* comb. nov. (al.gi’co.la. L. fem. n. *alga* alga; L. suff. *–cola* (from L. masc. n. *incola* inhabitant, dweller; N.L. masc. n. *algicola* algae-dweller)	*Alkalihalobacillus algicola* (Ivanova et al., 2004) Patel and Gupta, 2020	The description of this taxon is as given by Ivanova et al. (2004)	KMM 3737^T^ (= CIP 107850)
*Pseudalkalibacillus berkeleyi* comb. nov. (ber’ke.ley.i. N.L. *berkeleyi* is named after Roger C. W. Berkeley (1937– 2010), who is the famous English microbiologist who greatly contributed to the *Bacillus* taxonomy)	*Alkalihalobacillus berkeleyi* (Nedashkovskaya et al., 2012) Patel and Gupta, 2020	The description of this taxon is as given by Nedashkovskaya et al. (2012)	KMM 6244^T^ (= KCTC 12718 = LMG 26357)
**Description of the new combinations in the genus *Alkalicoccobacillus***			
*Alkalicoccobacillus murimartini* comb. nov. (Type species of this genus) (mu.ri.mar.ti’ni. L. masc. n. *murus* wall; N.L. gen. n. *martini* of Martin (masc. name of a saint); N.L. gen. n. *murimartini* from the wall of the (St.) Martin church in Greene-Kreiensen, Germany)	*Alkalihalobacillus murimartini* (Borchert et al., 2007) Patel and Gupta, 2020	The description of this taxon is as given by Borchert et al. (2007)	NCIMB 14102^T^ (= LMG 21005)
**Description of the new combinations in the genus *Alkalihalophilus***			
*Alkalihalophilus pseudofirmus* comb. nov. (Type species of this genus) (pseu.do.fir’mus. Gr. adj. *pseudês* false; L. masc. adj. *firmus* strong, firm, and also specific epithet; N.L. masc. adj. *pseudofirmus* the false *firmus*, referring to physiological similarities to *Bacillus firmus*)	*Alkalihalobacillus pseudofirmus* (Nielsen et al., 1995) Patel and Gupta, 2020	The description of this taxon is as given by Nielsen et al. (1995)	PN-3^T^ (= DSM 8715 = ATCC 700159)
*Alkalihalophilus lindianensis* comb. nov. (lin.di.an.en’sis. N.L. masc. adj. *lindianensis* pertaining to Lindian, a county in Heilongjiang Province, China, the source of the sample from which the type strain was isolated)	*Alkalihalobacillus lindianensis* (Dou et al., 2016) Patel and Gupta, 2020	The description of this taxon is as given by Dou et al. (2016)	12-3^T^ (= CGMCC 1.12717 = DSM 26864)
*Alkalihalophilus marmarensis* comb. nov. (mar.ma.ren’sis. N.L. masc. adj. *marmarensis* pertaining to the region of Marmara, where the type strain was isolated)	*Alkalihalobacillus marmarensis* (Denizci et al., 2010) Patel and Gupta, 2020	The description of this taxon is as given by Denizci et al. (2010)	GMBE 72^T^ (= DSM 21297 = JCM 15719)

### Emended Description of the Genus *Alkalihalobacillus* (Patel and Gupta, 2020)

Members can be isolated from the guts of larvae, soil, and feces. Cells are rod shaped, Gram-stain positive, endospore forming, aerobic, and motile, and tolerate NaCl concentration up to 5–10% with optimum growth at 2% w/v. Growth occurs in the range 10–40°C, with optimum growth at 30°C; alkaliphilic with growth in the range of pH 8–10 with optimum growth at pH 9. The major isoprenoid quinone is MK-7. The major fatty acids are *iso-*C_15:0_, *anteiso-*C_15:0_, *anteiso-*C_17:0_, and *iso-*C_17:0_. The polar lipid profile contains diphosphatidylglycerol, phosphatidylglycerol, phosphatidylethanolamine, and unidentified phospholipids. *meso*-DAP is cell wall diamino acid. The DNA G + C content is 36.2–39.0 mol%.

The type species is *Alkalihalobacillus alcalophilus*.

### Taxonomic Note on *Alkalihalobacillus okuhidensis* (Li et al., 2002; Patel and Gupta, 2020)

Based on ANI and dDDH analysis, *Alkalihalobacillus okuhidensis* is not a distinct species as the differences between *Ahb. halodurans* and *Ahb. okuhidensis* represent intra-species divergence. It should be noted that *Ahb. halodurans* and *Ahb. okuhidensis* differ with regard to fatty acid content, G + C content, growth at pH 6 and 11, utilization of carbon sources, hydrolysis of hippurate, tween 20, tween 40, and tween 60 (Li et al., 2002). Though there are minor differences in *Ahb. halodurans* and *Ahb. okuhidensis* but not enough for delineating two species. Therefore, based on the physiological, chemotaxonomic, and genotypic analysis, we propose *Ahb. okuhidensis* as a heterotypic synonym of *Alkalihalobacillus halodurans*.

### Description of *Alkalihalobacterium* gen. nov.

*Alkalihalobacterium* [Al.ka.li.ha.lo.bac.te’ri.um. N.L. n. *alkali*, alkali (from Arabic article *al*, the; Arabic n. *qaliy*, ashes of saltwort); N.L. neut. n. *bacterium*, a small rod; N.L. neut. n. *Alkalihalobacterium*, bacterium living under alkaline-saline conditions].

Long rod-shaped cells, Gram-stain positive, aerobic, and endospore forming, have been isolated from Soda lake soil/sediment, motile or non-motile, tolerate NaCl concentration up to 11.6% with optimum growth at 2.5–3.5% (w/v). Growth occurs in the range 10–45°C, with optimum growth temperature in the range 28–37°C. All members of this genus are alkaliphilic with growth at pH 8–11 (optimum 9). The major isoprenoid quinone is MK-7. The polar lipid profile contains diphosphatidylglycerol, phosphatidylglycerol, phosphatidylethanolamine, aminophospholipid, and unidentified phospholipids. The major fatty acids are *iso-*C_15:0_, *anteiso-*C_15:0_, *iso-*C_14:0_, *iso-*C_16:0_, and *iso-*C_17:0_. *meso*-DAP is cell wall diamino acid. The DNA G + C content 35.1–37.5%.

The type species is *Alkalihalobacterium alkalinitrilicum*.

### Description of *Alkalihalobacterium elongatum* sp. nov.

*Alkalihalobacterium elongatum* (e.lon.ga’tum. L. neut. part. adj. *elongatum* elongated).

Cells are motile, long, and thick rod shaped. Size is 6.4–16.5 μm × 0.6–2.4 μm (l × w). Gram-stain positive, aerobic, and forming subterminal oval endospore. The strain produced faint cream colored, dry, flat colonies with rhizoidal margins on nutrient agar (pH 10) medium, obligately alkaliphilic, and growth occurs between pH 8–11 with an optimum at pH 10. The optimum temperature for growth is 37°C and can grow at 10–45°C. The range of NaCl concentration is 0–10.5% (w/v) with optimum of 3.5% (w/v). Oxidase and catalase positive. Able to hydrolyze casein and esculin. Nitrate is reduced to nitrite. Not able to hydrolyze starch, gelatin, urea, and tween 40. Citrate is not utilized; methyl red, H_2_S, acetoin, or indole are not produced. Leucine arylamidase, valine arylamidase, α chymotrypsin, acid phosphatase, naphthol-AS-BI-phosphohydrolase, α glucosidase, and ß glucosidase activities are present. Alkaline phosphatase, esterase, esterase lipase, cysteine arylamidase, b-galactosidase, and *N*-acetyl-b-glucosaminidase activities are present. Lipase (C14), trypsin, α galactosidase ß glucuronidase, α-mannosidase and a-fucosidase activities are absent in API ZYM. In BIOLOG GEN III plate, the strain is negative for dextrin, D-maltose, D-trehalose, D-cellobiose, sucrose, turanose, stachyose, D-raffinose, α-D-lactose, β-methyl-D-glucoside, D-salicin, *N-*acetyl-D-glucosamine, *N-*acetyl-β-D-mannosamine *N-*acetyl-D-galactosamine, *N-*acetyl neuraminic acid, D-mannose, inosine, D-sorbitol, D-arabitol, D-mannitol, myo-inositol, glycerol D-glucose-6-PO_4_, D-aspartic acid, D-serine, gelatin, Glycyl-L-proline, L-alanine, L-arginine, L-aspartic acid, L-glutamic acid, L-histidine, L-pyroglutamic acid L-serine, pectin, L-galactonic acid lactone, D-gluconic acid, mucic acid, qunic acid, D-saccharic acid, p-hydroxy-phenylacetic acid, methyl pyruvate, D-lactic acid methyl ester, L-lactic acid, citric acid, α-keto-glutaric acid, D-malic acid, L-malic acid, tween 40 bromo-succinic acid, Υ-amino-butryric acid, α-hydroxy-butyric acid, β-hydroxy-D, L-butyric acid, α-keto-butyric acid, acetoacetic acid, acetic acid, and formic acid. The major cellular fatty acids are *iso-*C_15:0_, *iso-*C_16:0_, *anteiso-*C_15:0_, and *iso-*C_17:0_. Phosphatidylethanolamine, phosphatidylglycerol, and diphosphatidylglycerol as major polar lipids.

The type strain, MEB199^T^ (= MCC 2982^T^ = CGMCC 1.17254^T^ = JCM 33704), was isolated from a sediment sample collected from alkaline Lonar Lake, India. The DNA G + C content of the type strain is 36.47 mol%. The GenBank accession numbers for the 16S rRNA gene and draft genome sequence of strain MEB199^T^ are KX171019 and WMKZ00000000, respectively.

### Description of *Halalkalibacterium* gen. nov.

*Halalkalibacterium* (Hal.al.ka.li.bac.te’ri.um. Gr. masc. n. *hals*, salt; Arabic n. *al-qalyi*, soda ash; N.L. neut. n. *bacterium*, a small rod; N.L. neut. n. *Halalkalibacterium*, bacterium living under alkaline saline conditions).

Rod shaped, Gram-stain-variable. Members are aerobic or facultative anaerobes; endospore forming; have been isolated from sediments of the South China Sea/hot spa area, Motile, Tolerate NaCl concentration up to 12% with optimum growth at 2% (w/v). Growth occurs in the range 15–60°C, with optimum growth temperature in the range 30–40°C. All members of this genus are alkaliphilic with growth in the range of pH 6–11 with optimum growth at pH (9–10). The predominant polar lipids are diphosphatidylglycerol, phosphatidylglycerol, and phosphatidylethanolamine. The major isoprenoid quinone is MK-7. The major fatty acids are *iso-*C_14:0_ and *anteiso-*C_15:0_. *meso*-DAP is cell wall diamino acid. The DNA G + C content is 40.76–43.9 mol%.

The type species is *Halalkalibacterium halodurans*.

### Description of *Halalkalibacter* gen. nov.

*Halalkalibacter* (Hal.al.ka.li.bac’ter. Gr. masc. n. *hals*, salt; Arabic n. *al-qalyi*, soda ash; N.L. masc. n. *bacter*, rod; N.L. masc. n. *Halalkalibacter* briny and alkaline media-loving rod-shaped cells).

Cells are rod shaped, Gram-stain-variable, endospore forming, motile or non-motile, Aerobic or facultative anaerobic in nature, have been isolated from mushroom compost, sediment sample from the sea, seawater, soda pond, saltpan/soil, tolerate NaCl concentration up to 12% with optimum growth at 3–7% (w/v). Growth occurs in the range 10–50°C, with optimum growth at 30–37°C. Alkaliphilic with growth in the range of pH 6.5–12.5 with optimum growth at pH (9–10). The major isoprenoid quinone are MK-5/MK-6/MK-7. The major fatty acids are *anteiso-*C_15:0_ and *iso-*C_15:0_, C_16:0_ and *anteiso-*C_17:0_, C_14:0_, C_16:1_ω7c-alcohol, C_16:0_. Polar lipids are diphosphatidylglycerol, phosphatidylglycerol, phosphatidylethanolamine with a minor amount of aminophospholipid, and one glycolipid. *meso*-DAP is cell wall diamino acid. The DNA G + C content is 34.4–46.3 mol%.

The type species is *Halalkalibacter krulwichiae.*

### Description of *Shouchella* gen. nov.

*Shouchella* (Shou.chel’la. N.L. fem. dim. n. *Shouchella*, named after Dr. Yogesh Shouche, an eminent Indian microbiologist and taxonomist who has made a significant contribution in the field of microbial systematics and genomics of extremophilic bacteria from various extreme environments).

Cells are rod shaped, Gram-stain positive, endospore-forming, motile, or non-motile. All members are aerobic with few facultative anaerobes, have been isolated from rhizosphere soil of sugarcane/perennial shrub *Atriplex lampa* or *Miscanthus sacchariflorus* or soil or sediment sample from saline-alkaline habitat and non-saline forest soil. All the members are moderately halophilic and can tolerate NaCl concentrations up to 22% with optimum growth at 5–10% (w/v). Growth occurs in the range of 4–50°C, with optimum growth at 25–35°C. All members of this genus are alkalitolerant with growth in the range of pH 6.5–11 with optimum growth at pH 8–9. The major isoprenoid quinone is MK-7. The major fatty acids are *iso-*C_15:0_, *anteiso-*C_15:0_, *iso-*C_17:0_, and *anteiso-*C_17:0_, *iso-*C_14:0_, C_16:0_, and C_12:0_. The polar lipid profile contains diphosphatidylglycerol, phosphatidylglycerol, phosphatidylethanolamine, and glycolipid. *meso*-DAP is cell wall diamino acid. The DNA G + C content is 39.7–54 mol%.

The type species is *Shouchella clausii*.

### Description of *Pseudalkalibacillus* gen. nov.

*Pseudalkalibacillus* (Pseud.al.ka.li.ba.cil’lus. Gr. masc. adj. *pseudês*, false; N.L. masc. n. *Alkalibacillus* a bacterial genus; N.L. masc. adj. *Pseudalkalibacillus* a false *Alkalibacillus* because it only tolerates alkaline pH but does not grow optimally at higher pH).

Cells are rod shaped, Gram-stain positive, or Gram variable, endospore forming, motile, or non-motile, aerobic, facultative anaerobes, or anaerobic, have been isolated from the mud a goldmine/mangrove sediment, sea urchin, seawater, and mural paintings. All the members can tolerate NaCl concentrations up to 11% with optimum growth at 3–6% (w/v). Growth occurs in the range 4–50°C with optimum growth at 25–40°C. Most of the members of this genus can tolerate pH in the range of 5–10 with optimum growth at pH 7–8 and, thus, are alkalitolerant in nature. The major isoprenoid quinone is MK-7. The major fatty acids are *anteiso-*C_15:0_, *anteiso-*C_17:0_, and C_16:1_ω7c alcohol. Diphosphatidylglycerol, phosphatidylglycerol, and phosphatidylethanolamine are the major polar lipids. *meso*-DAP is cell wall diamino acid. The DNA G + C content is 37–40.9 mol%.

The type species is *Pseudalkalibacillus decolorationis*.

### Description of *Alkalicoccobacillus* gen. nov.

*Alkalicoccobacillus* (Al.ka.li.coc.co.ba.cil’lus. N.L. n. *alkali*, alkali; from Arabic article *al*, the; from Arabic n. *qaly*, ashes of saltwort; Gr. masc. n. *kokkos* a berry; L. masc. n. *bacillus* a small rod; N.L. masc. n. *Alkalicoccobacillus*, a cocobacillary rod living in basic surroundings).

Cells are coccoid rod shaped, Gram-stain variable, endospore forming, and motile, isolated from mural paintings, discolored by microbial growths. It can tolerate NaCl concentrations up to 10% with optimum growth at 4–7% (w/v). Growth occurs in the range 5–40°C, with optimum growth at 25–37°C. The member is alkalitolerant, which grows in the pH range of 7–11 with optimum growth at pH 8. The major fatty acids are *anteiso-*C_15:0_ and *anteiso-*C_17:0_. The DNA G + C content is 39⋅8 mol%.

The type species is *Alkalicoccobacillus murimartini*.

### Description of *Alkalihalophilus* gen. nov.

*Alkalihalophilus* (Al.ka.li.ha.lo’phi.lus. N.L. n. *alkali*, alkali (from Arabic article *al* the; Arabic n. *qaliy* ashes of saltwort); Gr. masc. n. *hals* (gen. *halos*), salt; Gr. masc. adj. *philos* loving; N.L. masc. n. *Alkalihalophilus*, bacterium liking alkaline and saline environment).

Cells are rod shaped, Gram-stain positive, aerobic, endospore-forming, and motile, have been isolated from saline and alkaline soils, mushroom compost, and animal manure. It can tolerate NaCl concentrations up to 12% with optimum growth at 4% (w/v). Growth occurs in the range 10–45°C, with optimum growth at 37°C. All the members are obligate alkaliphilic in nature and can grow in the pH range of 8–12, and no growth is found at pH 7 with optimum growth at pH 9. The major fatty acids are *anteiso-*C_15:0_, *iso-*C_15:0_ and *anteiso-*C_17:0_. Diphosphatidylglycerol, phosphatidylethanolamine, and phosphatidylglycerol are the major polar lipids. *meso*-DAP is cell wall diamino acid. The DNA G + C content is 39.0–42.7 mol%.

The type species is *Alkalihalophilus pseudofirmus*.

## Data Availability Statement

The datasets presented in this study can be found in online repositories. The names of the repository/repositories and accession number(s) can be found below: https://www.ncbi.nlm.nih.gov/genbank/, WMKZ00000000.

## Author Contributions

AJ and TL designed the work. ST and AJ carry out the morphological, biochemical, physiological and molecular characterisation of novel strain and maintained the bacterial cultures. TL carried out the genomic data retrieval from databases, phylogenomic, phylogenetic data analysis, and calculated the genomic indices. NJ carried out the FAME analysis and PK did the polar lipids profiling. All authors contributed to writing the manuscript and accepted it for publication.

## Conflict of Interest

The authors declare that the research was conducted in the absence of any commercial or financial relationships that could be construed as a potential conflict of interest.

## Publisher’s Note

All claims expressed in this article are solely those of the authors and do not necessarily represent those of their affiliated organizations, or those of the publisher, the editors and the reviewers. Any product that may be evaluated in this article, or claim that may be made by its manufacturer, is not guaranteed or endorsed by the publisher.

## Footnotes

1
https://lpsn.dsmz.de/

2
https://rast.nmpdr.org/

3
https://www.genome.jp/kegg/ko.html

4
http://www.heatmapper.ca/

5
https://www.ebi.ac.uk/Tools/msa/clustalo/

6
http://enve-omics.ce.gatech.edu/ani/

7
http://enve-omics.ce.gatech.edu/g-matrix/

8
http://ggdc.dsmz.de/
