# Corrigendum: *Alkalihalobacterium elongatum* gen. nov. sp. nov.: An Antibiotic-Producing Bacterium Isolated From Lonar Lake and Reclassification of the Genus *Alkalihalobacillus* Into Seven Novel Genera

**DOI:** 10.3389/fmicb.2022.871596

**Published:** 2022-03-23

**Authors:** Amaraja Joshi, Sonia Thite, Prachi Karodi, Neetha Joseph, Tushar Lodha

**Affiliations:** National Centre for Microbial Resource, National Centre for Cell Science, Pune, India

**Keywords:** alkaliphilic bacteria, *Alkalihalobacterium*, *Alkalihalobacillus*, taxogenomic, Lonar lake, *Shouchella*, *Halalkalibacterium*, *Halalkalibacter*

In the original article, there was a mistake in the description of the new combinations in the newly proposed genus (Table 5). Culture collection number for *Alkalihalobacterium bogoriense* was given as MG 22234 in the original manuscript. It is changed to LMG 22234. The strain number for the type strain *Alkalihalobacillus okhensis* was incorrect. The corrected Table 5 appears below.

**Table 5:** Description of the new combinations in the newly proposed genus.

**New Name combination and etymology**	**Basonym**	**Description**	**Type strain**
**Description of the new combinations in the genus** ***Alkalihalobacterium***			
*Alkalihalobacterium alkalinitrilicum* comb. nov. (Type species of this genus) (al.ka.li.ni.tri'li.cum. N.L. n. *alkali* (from Arabic *al* the; *qaliy* soda ash) alkali; N.L. masc. adj. *nitrilicus* pertaining to nitriles; N.L. neut. adj. *alkalinitrilicum* alkaliphile utilizing nitriles)	*Bacillus alkalinitrilicus* (Sorokin et al., 2008) Patel and Gupta, 2020	The description of this taxon is as given by Sorokin et al. (2008)	ANL-iso4^T^ (=DSM 22532= NCCB 100120 = UNIQEM U240)
*Alkalihalobacterium bogoriense* comb. nov. (bo.gor.i.en′se. N.L. neut. adj. *bogoriense* pertaining to Lake Bogoria, a soda lake in Kenya)	*Alkalihalobacillus bogoriensis* (Vargas et al., 2005) Patel and Gupta, 2020	The description of this taxon is as given by Vargas et al. (2005)	LBB3^T^ (=ATCC BAA-922 = LMG 22234)
**Description of the new combinations in the genus** ***Halalkalibacterium***			
*Halalkalibacterium halodurans* comb. nov. (Type species of this genus) (ha.lo.du'rans. Gr. n. *hals halos* salt; L. pres. part. *durans* enduring; N.L. part. adj. *halodurans* salt-enduring)	*Alkalihalobacillus halodurans* (ex Boyer et al., 1973) (Nielsen et al., 1995) Patel and Gupta, 2020	The description of this taxon is as given by Nielsen et al. (1995)	PN-80^T^ (=ATCC 27557 = CIP 105296 = DSM 497 = LMG 7121 = NRRL B-3881)
*Halalkalibacterium ligniniphilum* comb. nov. (lig.ni.ni'phi.lum. N.L. neut. n. *ligninum* lignin; N.L. masc. adj. *philus* (from Gr. masc. adj. *philos*) friend, loving; N.L. neut. adj. *ligniniphilum* lignin-loving, isolated as a lignin degrader with lignin as a single carbon source)	*Alkalihalobacillus ligniniphilus* (Zhu et al., 2014) Patel and Gupta, 2020	The description of this taxon is as given by Zhu et al. (2014)	L1^T^ (= DSM 26145 = JCM 18543)
**Description of the new combinations in the genus** ***Halalkalibacter***			
*Halalkalibacter krulwichiae* comb. nov. (Type species of this genus) (krul.wich'i.ae. N.L. fem. gen. n. *krulwichiae* of Krulwich; named after American microbiologist Terry A. Krulwich who made fundamental contributions to the study of alkaliphilic bacteria)	*Alkalihalobacillus krulwichiae* (Yumoto et al., 2003) Patel and Gupta, 2020	The description of this taxon is as given by Yumoto et al. (2003)	AM31D^T^ (=DSM 18225 = IAM 15000 = JCM 11691 = NBRC 102362 = NCIMB 13904)
*Halalkalibacter oceani* comb. nov. (o.ce.a'ni. L. gen. masc. n. *oceani*, of an ocean, referring to its optimal growth under marine conditions)	*Alkalihalobacillus oceani* (Song et al., 2016) Gupta et al., 2020	The description of this taxon is as given by Song et al. (2016)	SW109^T^ (=CGMCC 1.12347 = DSM 100579)
*Halalkalibacter hemicellulosilyticus* comb. nov. (he.mi.cel.lu.lo.si.ly'ti.cus N.L. neut. n. *haemicellulosum*, hemicellulose; Gr. masc. adj. *lytikos*, able to loosen, able to dissolve; N.L. masc. adj. *hemicellulosilyticus*, hemicellulose-dissolving)	*Alkalihalobacillus hemicellulosilyticus* corrig. (Nogi et al., 2005) Patel and Gupta, 2020	The description of this taxon is as given by Nogi et al. (2005)	C-11^T^ (=DSM 16731 = JCM 9152)
*Halalkalibacter nanhaiisediminis* comb. nov. (nan.hai.i.se.di'mi.nis. N.L. neut. n. *nanhaium* Nan Hai, the Chinese name for the South China Sea; L. neut. n. *sedimen -inis* a sediment; N.L. gen. n. *nanhaiisediminis* of a sediment from the South China Sea)	*Alkalihalobacillus nanhaiisediminis* (Zhang et al., 2011) Patel and Gupta, 2020	The description of this taxon is as given by Zhang et al. (2011)	NH3^T^ (=CGMCC 1.10116 = DSM 27953 = JCM 16507)
*Halalkalibacter wakoensis* comb. nov. (wa.ko.en'sis. N.L. masc. adj. *wakoensis* of Wako, a city in Japan)	*Alkalihalobacillus wakoensis* (Nogi et al., 2005) Patel and Gupta, 2020	The description of this taxon is as given by Nogi et al. (2005)	N-1^T^ (=DSM 2521 = JCM 9140)
*Halalkalibacter okhensis* comb. nov. (ok.hen'sis. N.L. masc. adj. *okhensis* pertaining to Port Okha, a port of the Dwarka region in India, where the type strain was isolated)	*Alkalihalobacillus okhensis* (Nowlan et al., 2006) Patel and Gupta, 2020	The description of this taxon is as given by Nowlan et al. (2006)	Kh10-101^T^ (=ATCC BAA-1137 = DSM 23308 = JCM 13040)
*Halalkalibacter akibai* comb. nov. (a.ki.ba'i. N.L. gen. n. *akibai* of Akiba, named after the Japanese microbiologist Teruhiko Akiba, who made fundamental contributions to the study of alkaliphilic bacteria)	*Alkalihalobacillus akibai* (Nogi et al., 2005) Patel and Gupta, 2020	The description of this taxon is as given by Nogi et al. (2005)	1139^T^ (=ATCC 43226 = DSM 21942 = JCM 9157)
*Halalkalibacter kiskunsagensis* comb. nov. (kis.kun.sag.en'sis N.L. masc. adj. *kiskunsagensis*, referring to the name of Kiskunság National Park in Hungary, the location of the sampling site)	*Alkalihalobacillus kiskunsagensis* (Borsodi et al., 2017) Gupta et al., 2020	The description of this taxon is as given by Borsodi et al. (2017)	B16-24^T^ (=DSM 29791 = NCAIM B.02610)
*Halalkalibacter urbisdiaboli* comb. nov. (ur.bis.di.a'bo.li. L. fem. n. *urbs*, city; L. masc. n. *diabolus*, devil; N.L. gen. n. *urbisdiaboli*, of Devil City)	*Alkalihalobacillus urbisdiaboli* (Liu et al., 2019) Gupta et al., 2020	The description of this taxon is as given by Liu et al. (2019)	FJAT-45385^T^ (=CCTCC AB 2016263 = DSM 104651)
*Halalkalibacter alkalisediminis* comb. nov. (al.ka.li.se.di′mi.nis. N.L. n. *alkali* (from Arabic *al* the; *qaliy* soda ash) alkali; L. gen. n. *sediminis* of sediment; N.L. gen. n. *alkalisediminis* of alkaline sediment)	*Alkalihalobacillus alkalisediminis* (Borsodi et al., 2011) Patel and Gupta, 2020	The description of this taxon is as given by Borsodi et al. (2011)	K1-25^T^ (=DSM 21670 = NCAIM B.02301)
**Description of the new combinations in the genus** ***Shouchella***			
*Shouchella clausii* comb. nov. (Type species of this genus) (clau′si.i. N.L. gen. n. *clausii* of Claus, named after Dieter Claus, the German bacteriologist who made fundamental contributions to the taxonomy of *Bacillus*)	*Alkalihalobacillus clausii* (Nielsen et al., 1995) Patel and Gupta, 2020	The description of this taxon is as given by Nielsen et al. (1995)	PN-23^T^ (=ATCC 700160 = CCUG 47262 = CIP 104718 = DSM 8716 = LMG 17945 = NCIB 10309 = NCIMB 10309)
*Shouchella xiaoxiensis* comb. nov. (xi.a.o.xi.en′sis. N.L. fem. adj. *xiaoxiensis* pertaining to Xiaoxi National Natural Reserve, China, the source of the sample from which the type strain was isolated)	*Alkalihalobacillus xiaoxiensis* (Chen et al., 2011b) Patel and Gupta, 2020	The description of this taxon is as given by Chen et al. (2011b)	DSM 21943^T^ (=JSM 081004 = CCTCC AA 208057)
*Shouchella alkalilacus* comb. nov. (al.ka.li.la'cus. N.L. n. *alkali* (from Arabic *al* the; *qaliy* soda ash) alkali; L. gen. masc. n. *lacus*, a lake; N.L. gen. masc. n. *alkalilacus*, of an alkaline lake)	*Alkalihalobacillus alkalilacus* (Singh et al., 2018) Gupta et al., 2020	The description of this taxon is as given by Singh et al. (2018)	AK73^T^ (=JCM 32184 = KCTC 33880 = MTCC 12637)
*Shouchella rhizosphaerae* comb. nov. (rhi.zo.sphae'rae. N.L. gen. n. *rhizosphaerae* of the rhizosphere)	*Alkalihalobacillus rhizosphaerae* (Madhaiyan et al., 2011) Patel and Gupta, 2020	The description of this taxon is as given by Madhaiyan et al. (2011)	SC-N012^T^ (=DSM 21911 = NCCB 100267)
*Shouchella patagoniensis* comb. nov. (pa.ta.go.ni.en'sis. N.L. fem. adj. *patagoniensis* pertaining to Patagonia, in Argentina, where the type strain was isolated)	*Alkalihalobacillus patagoniensis* (Olivera et al., 2005) Patel and Gupta, 2020	The description of this taxon is as given by Olivera et al. (2005)	PAT 5^T^ (=ATCC BAA-965 = DSM 16117)
*Shouchella miscanthi* comb. nov. (misc.an'thi. N.L. gen. *masc*. n. *miscanthi*, of *Miscanthus sacchariflorus*, where the type strain was isolated)	*Alkalihalobacillus miscanthi* (Shin et al., 2020) Gupta et al., 2020	The description of this taxon is as given by Shin et al. (2020)	AK13^T^ (= DSM 109981 = KACC 21401)
*Shouchella plakortidis* comb. nov. (pla.kor′ti.dis. N.L. gen. n. *plakortidis* of *Plakortis*, a genus of sponges)	*Alkalihalobacillus plakortidis* (Borchert et al., 2007) Patel and Gupta, 2020	The description of this taxon is as given by Borchert et al. (2007)	P203^T^ (=DSM 19153 = NCIMB 14288)
*Shouchella oshimensis* comb. nov. (o.shi.men'sis. N.L. fem. adj. *oshimensis* from Oshima, the region where the micro-organism was isolated)	*Alkalihalobacillus oshimensis* (Yumoto et al., 2005) Patel and Gupta, 2020	The description of this taxon is as given by Yumoto et al. (2005)	K11 ^T^(=DSM 18940 = JCM 12663 = NCIMB 14023)
*Shouchella lehensis* comb. nov. (le.hen'sis. N.L. fem. adj. *lehensis* pertaining to Leh, in India, where the type strain was isolated)	*Alkalihalobacillus lehensis* (Ghosh et al., 2007) Patel and Gupta, 2020	The description of this taxon is as given by Ghosh et al. (2007)	MTCC 7633^T^ (=MLB2 = JCM 13820 = LMG 24751 = DSM 19099)
*Shouchella shacheensis* comb. nov. (sha.che.en'sis. N.L. fem. adj. *shacheensis* pertaining to Shache County, Xinjiang Province, China, the source of the sample from which the type strain was isolated)	*Alkalihalobacillus shacheensis* (Lei et al., 2014) Patel and Gupta, 2020	The description of this taxon is as given by Lei et al. (2014)	HNA-14^T^ (=DSM 26902 = KCTC 33145)
*Shouchella lonarensis* comb. nov. (lo.nar.en′sis. N.L. fem. adj. *lonarensis* of or belonging to Lonar lake, India, from where the type strain was isolated).	*Alkalihalobacillus lonarensis* (Reddy et al., 2015) Patel and Gupta, 2020	The description of this taxon is as given by Reddy et al. (2015)	LMG 27974^T^ (=CGMCC 1.12817 = KCTC 33413 = 25 nlg)
*Shouchella hunanensis* comb. nov. (hu.nan.en'sis. N.L. fem. adj. *hunanensis*, pertaining to Hunan Province, PR China, the source of the sample from which the type strain was isolated)	*Alkalihalobacillus hunanensis* Patel and Gupta, 2020	The description of this taxon is as given by Patel and Gupta (2020)	JSM 081003^T^ (=DSM 23008 = KCTC 13711)
*Shouchella gibsoni* comb. nov. (gib.so'ni.i. N.L. gen. masc. n. *gibsonii*, of Gibson, named after the British bacteriologist Thomas Gibson)	*Alkalihalobacillus gibsonii* (Nielsen et al., 1995) Gupta et al., 2020	The description of this taxon is as given by Nielsen et al. (1995)	PN-109^T^ (=ATCC 700164 = CIP 104720 = DSM 8722 = LMG 17949)
**Description of the new combinations in the genus** ***Pseudalkalibacillus***			
*Pseudalkalibacillus decolorationis* comb. nov. (Type species of this genus) (de.co.lo.ra.ti.o'nis. L. gen. n. *decolorationis* of discoloration)	*Alkalihalobacillus decolorationis* (Heyrman et al., 2003) Patel and Gupta, 2020	The description of this taxon is as given by Heyrman et al. (2003)	LMG 19507^T^ (=DSM 14890)
*Pseudalkalibacillus macyae* comb. nov. (ma'cy.ae. N.L. fem. n. *macyae* of Macy, named after the late Professor Joan M. Macy, Chair of Microbiology, La Trobe University, in tribute to her research in the area of environmental microbiology)	*Alkalihalobacillus macyae* (Santini et al., 2004) Patel and Gupta (2020)	The description of this taxon is as given by Santini et al. (2004)	JMM-4^T^ (=DSM 16346 = JCM 12340)
*Pseudalkalibacillus caeni* comb. nov. (cae'ni. L. gen. neut. n. *caeni*, of mud, referring to the source of isolation)	*Alkalihalobacillus caeni* (Mo et al., 2020) Gupta et al., 2020	The description of this taxon is as given by Mo et al. (2020)	HB172195^T^(=CGMCC 1.16730 = JCM 33411)
*Pseudalkalibacillus hemicentroti* comb. nov. (he.mi.cen.tro'ti. N.L. gen. n. haemicentroti of Haemicentrotus (*Haemicentrotus pulcherrimus*, a sea urchin), the source of isolation of the organism)	*Alkalihalobacillus haemicentroti* (Chen et al., 2011a) Patel and Gupta, 2020	The description of this taxon is as given by Chen et al. (2011a)	JSM 076093^T^(=DSM 23007 = KCTC 13710)
*Pseudalkalibacillus hwajinpoensis* comb. nov. (hwa.jin.po.en'sis. N.L. masc. adj. *hwajinpoensis* of Hwajinpo, a beach of the East Sea in Korea, where the type strain was isolated)	*Alkalihalobacillus hwajinpoensis* (Yoon et al., 2004) Patel and Gupta, 2020	The description of this taxon is as given by Yoon et al. (2004)	SW-72^T^(=DSM 16206 = JCM 11807 = KCCM 41641 = LMG 24749)
*Pseudalkalibacillus algicola* comb. nov. (al.gi′co.la. L. fem. n. *alga* alga; L. suff. –*cola* (from L. masc. n. *incola* inhabitant, dweller; N.L. masc. n. *algicola* algae-dweller)	*Alkalihalobacillus algicola* (Ivanova et al., 2004) Patel and Gupta, 2020	The description of this taxon is as given by Ivanova et al. (2004)	KMM 3737^T^(=CIP 107850)
*Pseudalkalibacillus berkeleyi* comb. nov. (ber′ke.ley.i. N.L. *berkeleyi* is named after Roger C. W. Berkeley (1937–2010), who is the famous English microbiologist who greatly contributed to the *Bacillus* taxonomy)	*Alkalihalobacillus berkeleyi* (Nedashkovskaya et al., 2012) Patel and Gupta, 2020	The description of this taxon is as given by Nedashkovskaya et al. (2012)	KMM 6244^T^(=KCTC 12718 = LMG 26357)
**Description of the new combinations in the genus** ***Alkalicoccobacillus***			
*Alkalicoccobacillus murimartini* comb. nov. (Type species of this genus) (mu.ri.mar.ti′ni. L. masc. n. *murus* wall; N.L. gen. n. *martini* of Martin (masc. name of a saint); N.L. gen. n. *murimartini* from the wall of the (St.) Martin church in Greene-Kreiensen, Germany)	*Alkalihalobacillus murimartini* (Borchert et al., 2007) Patel and Gupta, 2020	The description of this taxon is as given by Borchert et al. (2007)	NCIMB 14102^T^ (=LMG 21005)
**Description of the new combinations in the genus** ***Alkalihalophilus***			
*Alkalihalophilus pseudofirmus* comb. nov. (Type species of this genus) (pseu.do.fir′mus. Gr. adj. *pseudês* false; L. masc. adj. *firmus* strong, firm, and also specific epithet; N.L. masc. adj. *pseudofirmus* the false *firmus*, referring to physiological similarities to *Bacillus firmus*)	*Alkalihalobacillus pseudofirmus* (Nielsen et al., 1995) Patel and Gupta (2020)	The description of this taxon is as given by Nielsen et al. (1995)	PN-3^T^ (=DSM 8715 = ATCC 700159)
*Alkalihalophilus lindianensis* comb. nov. (lin.di.an.en'sis. N.L. masc. adj. *lindianensis* pertaining to Lindian, a county in Heilongjiang Province, China, the source of the sample from which the type strain was isolated)	*Alkalihalobacillus lindianensis* (Dou et al., 2016) Patel and Gupta, 2020	The description of this taxon is as given by Dou et al. (2016)	12-3^T^ (=CGMCC 1.12717 = DSM 26864)
*Alkalihalophilus marmarensis* comb. nov. (mar.ma.ren′sis. N.L. masc. adj. *marmarensis* pertaining to the region of Marmara, where the type strain was isolated)	*Alkalihalobacillus marmarensis* (Denizci et al., 2010) Patel and Gupta, 2020	The description of this taxon is as given by Denizci et al. (2010)	GMBE 72^T^ (=DSM 21297 = JCM 15719)

In the original article, there was an error in the naming of a family species, *Bacilla* was written where it should be *Bacillaceae*. A correction has been made to **Materials and Methods**, **Genome Sequences Used in This Study and Pathway Analysis**, paragraph one:

“Type strains of 41 species of the genus *Alkalihalobacillus* (28 type strains), *Desertibacillus*, and *Anaerobacillus* and type species of the different genera of the family ***Bacillaceae***, whose genomes were available in the public database, were considered in this study. Fifty genomes of non-type strains of genus *Alkalihalobacillus* were also included in this study. *Streptococcus gordonii* ATCC 10558^T^ and *Streptococcus agalactiae* ATCC 13813^T^ genomes were used to root (outgroup) the phylogenetic tree. The genome of strain MEB199^T^ and *Desertibacillus haloalkaliphilus* KJ1-10-99^T^ were sequenced under this study, and all the other genomes were downloaded from the PATRIC database (Supplementary Table 1). The functional annotations of each genome was carried out using EggNOG-mapper v2 (Cantalapiedra et al., 2021). The pathway was mapped using the KEGG Orthology (KO) Database^3^ for all the selected genomes. Heatmap to visualize the distribution of pathways across all the members of the genus *Alkalihalobacillus* was constructed using heatmapper.^4^”

The authors apologize for these errors and state that this does not change the scientific conclusions of the article in any way.

## Publisher's Note

All claims expressed in this article are solely those of the authors and do not necessarily represent those of their affiliated organizations, or those of the publisher, the editors and the reviewers. Any product that may be evaluated in this article, or claim that may be made by its manufacturer, is not guaranteed or endorsed by the publisher.

## Footnotes

^3^ https://www.genome.jp/kegg/ko.html

^4^ http://www.heatmapper.ca/
