# Supplementary material for: Alkalihalobacterium elongatum gen. nov. sp. nov.: An Antibiotic-Producing Bacterium Isolated From Lonar Lake and Reclassification of the Genus Alkalihalobacillus Into Seven Novel Genera
Source: Front Microbiol. 2021 Oct 11;12:722369. doi: 10.3389/fmicb.2021.722369 (PMC8543038; doi:10.3389/fmicb.2021.722369)
Supplement: Supplementary file 1 [file Image_1.PDF]

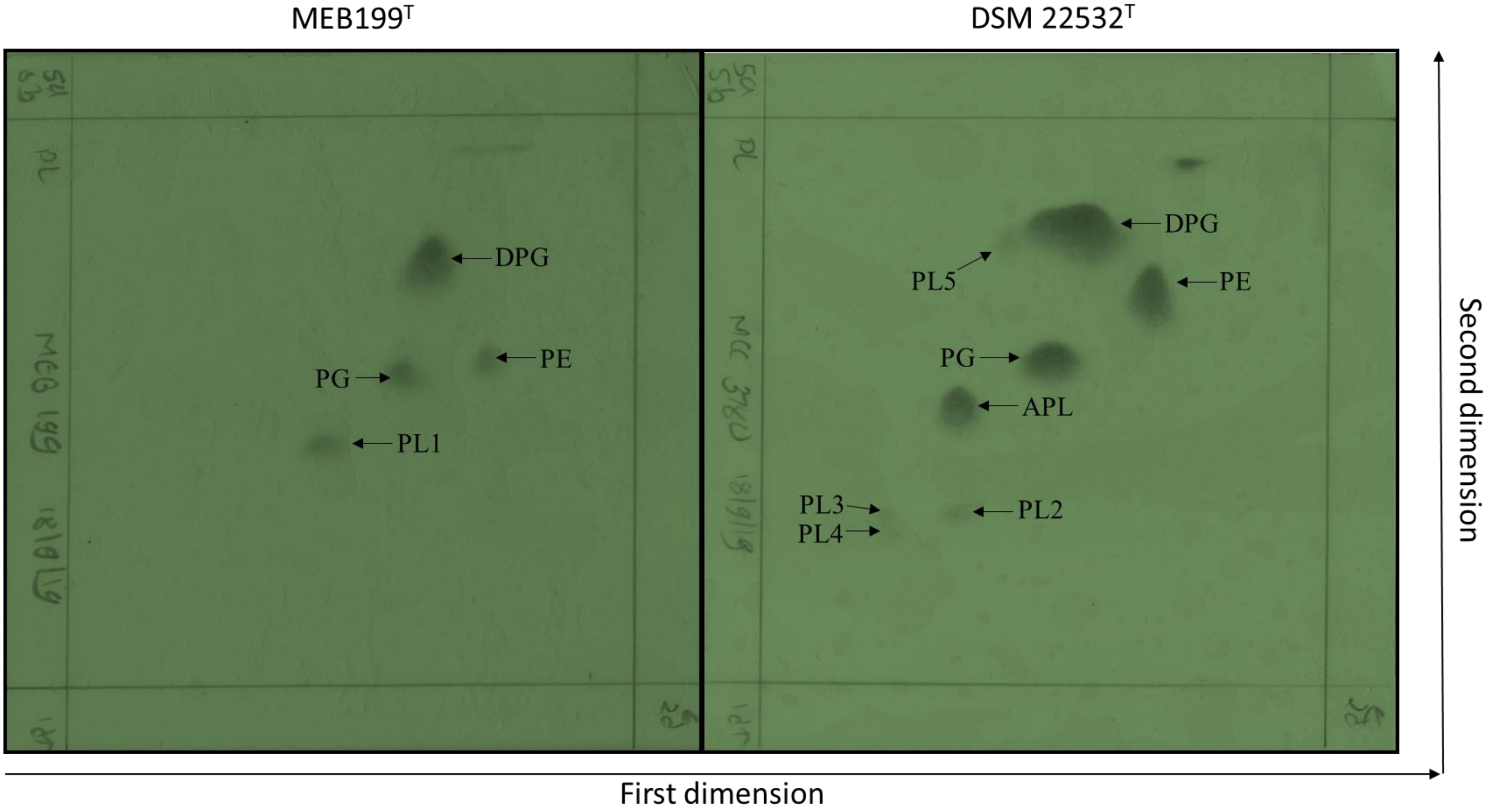

Supplementary Figure S1. Two-dimensional thin-layer chromatogram of whole cell lipid extracts of strain MEB199^T and *Alkalihalobacillus alkalinitrilicus* DSM 22532^T. The first direction was developed in chloroform-methanol-water (65:25:4 by vol.) and the second in chloroform-acetic acid-methanol-water (40:7.5:6:2, by vol.). DPG, Diphosphatidylglycerol; PE, Phosphatidylethanolamine; PG, Phosphatidylglycerol; PL, Unidentified phospholipid lipid; APL, Unidentified amino phospholipid.
