# Supplementary material for: Alkalihalobacterium elongatum gen. nov. sp. nov.: An Antibiotic-Producing Bacterium Isolated From Lonar Lake and Reclassification of the Genus Alkalihalobacillus Into Seven Novel Genera
Source: Front Microbiol. 2021 Oct 11;12:722369. doi: 10.3389/fmicb.2021.722369 (PMC8543038; doi:10.3389/fmicb.2021.722369)
Supplement: Supplementary file 2 [file Image_2.PDF]

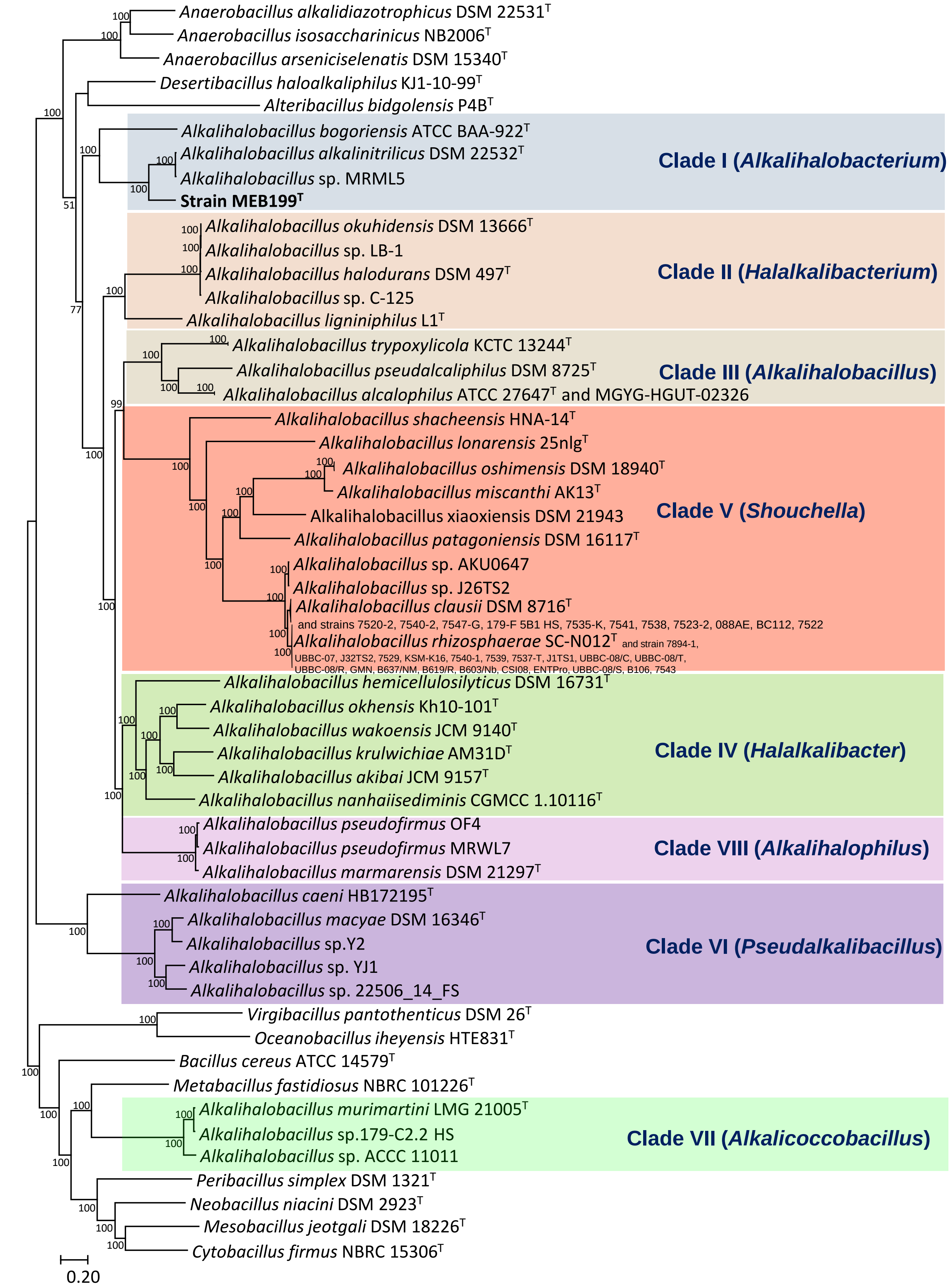

Supplementary Figure S2. A codon tree based on single-copy genes was reconstructed using amino acid and nucleotide sequences showing the relationships of the members of genus *Alkalihalobacillus* and nearest genera. The tree was constructed using PATRIC server. Bar, 0.2 nucleotide substitution per position.
