# Supplementary material for: Alkalihalobacterium elongatum gen. nov. sp. nov.: An Antibiotic-Producing Bacterium Isolated From Lonar Lake and Reclassification of the Genus Alkalihalobacillus Into Seven Novel Genera
Source: Front Microbiol. 2021 Oct 11;12:722369. doi: 10.3389/fmicb.2021.722369 (PMC8543038; doi:10.3389/fmicb.2021.722369)
Supplement: Supplementary file 3 [file Image_3.PDF]

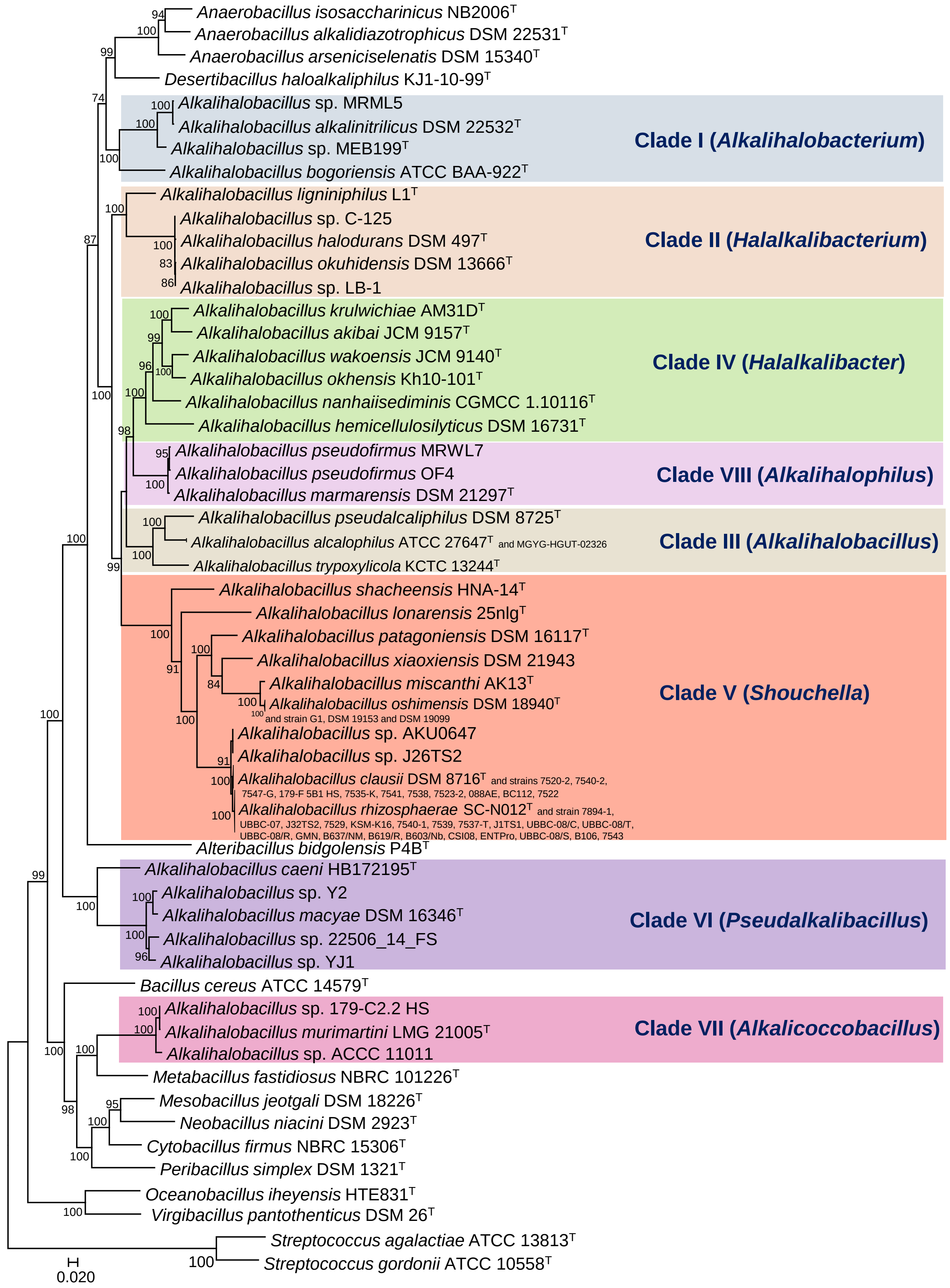

Supplementary Figure S3. Phylogenetic tree constructed using the 120 ubiquitous single-copy proteins showing the relationships of the members of genus *Alkalihalobacillus* and nearest genera. The tree was constructed using Genome Taxonomy Database toolkit (GTDB-Tk). Bar, 0.02 nucleotide substitution per position.
