# Supplementary material for: Alkalihalobacterium elongatum gen. nov. sp. nov.: An Antibiotic-Producing Bacterium Isolated From Lonar Lake and Reclassification of the Genus Alkalihalobacillus Into Seven Novel Genera
Source: Front Microbiol. 2021 Oct 11;12:722369. doi: 10.3389/fmicb.2021.722369 (PMC8543038; doi:10.3389/fmicb.2021.722369)
Supplement: Supplementary file 4 [file Image_4.PDF]

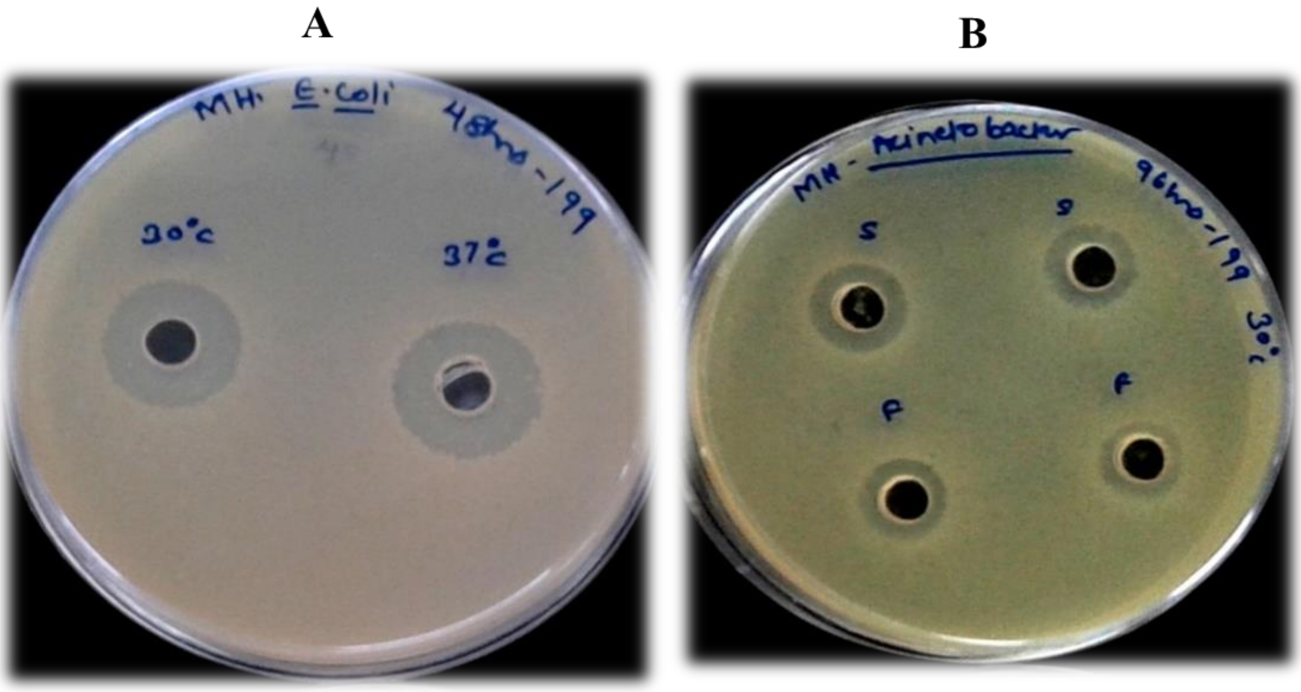

Supplementary Figure S4. Antimicrobial activity of the concentrated supernatant of the strain MEB199^T showing the inhibition zone against (A) *E. coli* BAC03 and (B) *Acinetobacter baumannii* BAC01
