# Supplementary material for: Alkalihalobacterium elongatum gen. nov. sp. nov.: An Antibiotic-Producing Bacterium Isolated From Lonar Lake and Reclassification of the Genus Alkalihalobacillus Into Seven Novel Genera
Source: Front Microbiol. 2021 Oct 11;12:722369. doi: 10.3389/fmicb.2021.722369 (PMC8543038; doi:10.3389/fmicb.2021.722369)
Supplement: Supplementary file 5 [file Image_5.PDF]

```
                                                      172                                                    226
Alkalihalobacillus lonarensis                 NMLVIWLDYEEG-DSYFEEAQKQMQGEEPQFLSAARVQEPIHSTNVMIE--GN-FTTEET
Alkalihalobacillus clausii                    NLLVIWLDFEEG-DSFAEESKK----QDPKYMSAASVNAPLHTRDVMIE--GN-FTTEET
Alkalihalobacillus rhizosphaerae              NLLVIWLDFEEG-DSFAEESKK----QDPKYMSAASVNAPLHTRDVMIE--GN-FTTEET
Alkalihalobacillus patagoniensis              NLLVMWLDFEEG-DSYEEERLK----EDPKFLSAASVRQPLFTRNVMIE--GN-FTTEET
Alkalihalobacillus oshimensis                 NLLVMWLDFEEG-DSYEEEAMK----ADPKFLSAASVRQTLQTRNVQIE--GQ-FTTEET
Alkalihalobacillus lehensis                   NLLVMWLDFEEG-DSYEEEAMK----ADPKFLSAASVRQTLQTRNVQIE--GQ-FTTEET
Alkalihalobacillus plakortidis                NLLVMWLDFEEG-DSYEEEAMK----ADPKFLSAASVRQTLQTRNVQIE--GQ-FTTEET
Alkalihalobacillus miscanthi                  NLLVMWLDFEEG-DSYEEEAMK----ADPKFLSAASVRQTLQTRNVQIE--GQ-FTTEET
Alkalihalobacillus macyae                     NRLVIWLDYEEG-DSYQEEVTKP--ADEQKFISDPAVRTVLSQNTVVIS--GPPFQLEEA
Alkalihalobacillus caeni                      NRLVIWLDYEKG-DSYKEELKKP--EGERKFISDPQVTQVLSQNQVIIT--GPPFKVEEA
Alteribacillus bidgolensis                    NRLVIWLDYEEG-DSFAEEYQQE----EPKYLSAPAVNQPLYHKNIQIE---GNFELEEA
Alkalihalobacillus alcalophilus               NLMVIWLDFEEG-DSYYGEAMKE----EPKFLSAATVGEEFRTTTVQIE--G-TFSLDEA
Alkalihalobacillus pseudalcaliphilus          NLMVIWLDFEDG-DSFYGETGKG----DPKFLSAATVGEVFRTTQVQI---TGDFTMNEA
Anaerobacillus alkalidiazotrophicus           SQLVIWLDYQES-DSFMEEMLKP--EHERKFISAPTVRSVLNTTNVVIE--G-TFTLEEA
Anaerobacillus arseniciselenatis              SQLVIWLDYEDG-DSFMEERLKP--EAEQKFISAPSVSEVLNTRNVRI---TGNFTLAEA
Anaerobacillus isosaccharinicus               SQLVIWMDYEEG-DSFMEERLKP--ESEQKFISAPSVDQVLNTTNVVIR--G-TFTLEEA
Alkalihalobacillus bogoriensis                NRLVIWLDYEEG-DSYYEEAMKA----EPKFISDASVRAPINSRNVMIE--SSTFTQQQT
Alkalihalobacillus ligniniphilus              NLLVIWLDFEEGVDSFAEEALKP----DPKYISAASVTQTLNTRDVMIESRGGGFTVEET ┐
Alkalihalobacillus okuhidensis                NLLVIWLDFEEGVDSYAEEAMKA----SPKYISAASVNQPLFTRDVMIENRSGDFTVEQT ├ Clade II
Alkalihalobacillus halodurans                 NLLVIWLDFEEGVDSYAEEAMKA----SPKYISAASVNQPLFTRDVMIENRSGDFTVEQT ┘
Alkalihalobacillus hemicellulosilyticus       NMLVVWLDFEEGVDSYEEERMKA----DPKYLSAAVVRDVITSPTATI---SGDFTVEET
Alkalihalobacillus trypoxylicola              NLLVIWLDFEEGVDSYQEEVMKE----EPKFLSAASVDRVIDSRDVQIT--GPNFTVEET
Strain MEB199                                 NLLVIWLDYEEG-DSFYEEVTKE----NPKFLSAPTVREVLNTRNVVIQ--GS-FTIDEA
Alkalihalobacillus alkalinitrilicus           NLLVIWLDYEEG-DSFFEEAQKE----APKFLSAPTVRQVLNTTNVVIQ--GS-FTIEEA
Desertibacillus haloalkaliphilus              NLLVIWLDYEEG-DSFQEELMKE----EPKFMSAPRVGQTLNTRNVMIE--GS-FTLEET
Alkalihalobacillus marmarensis                NLLVIWLDFEEGVDSYEAEMMSE----DPKFLSAASVRQVIPSRDVMIE--GN-FTVEET
Alkalihalobacillus nanhaiisediminis           NLLVIWLDFEEGVDSYANEAAKE----DPKFLSAAVVRQVIPSNTATI---SGSFTLEET
Alkalihalobacillus akibai                     NLLVMWLDFEEGVDSYAAEAMKA----DPKYLSAARVVEVIPGNTATIT--GS-FTVDQT
Alkalihalobacillus okhensis                   NLLVMWLDFEEGVDSYAEEALKE----DPMYLSAATVREIIFSNTATI---SGNFTLEQT
Oceanobacillus iheyensis                      SRLVIWMDYQEG-DSFEEEAAKA----EPKYVSAPNVDGPINSSNVMI---NGNFTVESA
Virgibacillus pantothenticus                  NLLVIWMDYKKG-DSYAEEYQKD----EPKFVSAPSVNQTLNTSQVMI---SGNFTVESA
Alkalihalobacillus ligniniphilus              NLLVIWLDYEEG-DSYAEEAQKA----EPKFLSAATVRQKLNTTDVVIE--GQ-FTMKEA
Alkalihalobacillus murimartini                NQLVIWLDYDKG-DSFKKEVQKE----HPKFVSAPNVSQELNTTDVKIE--GH-FTAQEA
Metabacillus fastidiosus                      NQLVIWLDYQEG-DSFQKEIAKP----NPKFISAPNVGQVFNQTDVSI---TGQFTVKEA
Bacillus cereus                               NLMVIWLDFEEGKDSYKAESAKP----NPKFLSAATVNQVFNQAEVSIV--GGNFTVESA
Peribacillus simplex                          NVLAIWLDFEEGKDSIKDTA--S----QDNMISAPAVSEVFNTKKVYI---TGQFTVEEA
Mesobacillus jeotgali                         NQLIIWLDFEEGKDSYKEERTKP----EPKFLSNPNVDKILNQKNVEI---TGNFTIEEA
Cytobacillus firmus                           NLLVIWLDFEEGQDSFQAEAAKK----DPKYLSAPQVSQIFNQDTVSI---VGNFTIEEA
Neobacillus niacini                           NYLVIWLDFEEGKDSFKTEIAKE----DPKFLSAPTVREIFNQNTVSIV--GS-FTAEEA
                                              . : :*:*:... **            :*   *    :      *      *   .:
```

Supplementary Figure S5. A partial sequence alignment of amino acid sequence of the translocase subunit (secD) protein containing a amino acid insertion (boxed) that is exclusively shared by all members of the Clade II containing a homolog of this protein and absent in other members of the genus *Alkalihalobacillus.*
