# Supplementary material for: Alkalihalobacterium elongatum gen. nov. sp. nov.: An Antibiotic-Producing Bacterium Isolated From Lonar Lake and Reclassification of the Genus Alkalihalobacillus Into Seven Novel Genera
Source: Front Microbiol. 2021 Oct 11;12:722369. doi: 10.3389/fmicb.2021.722369 (PMC8543038; doi:10.3389/fmicb.2021.722369)
Supplement: Supplementary file 6 [file Image_6.PDF]

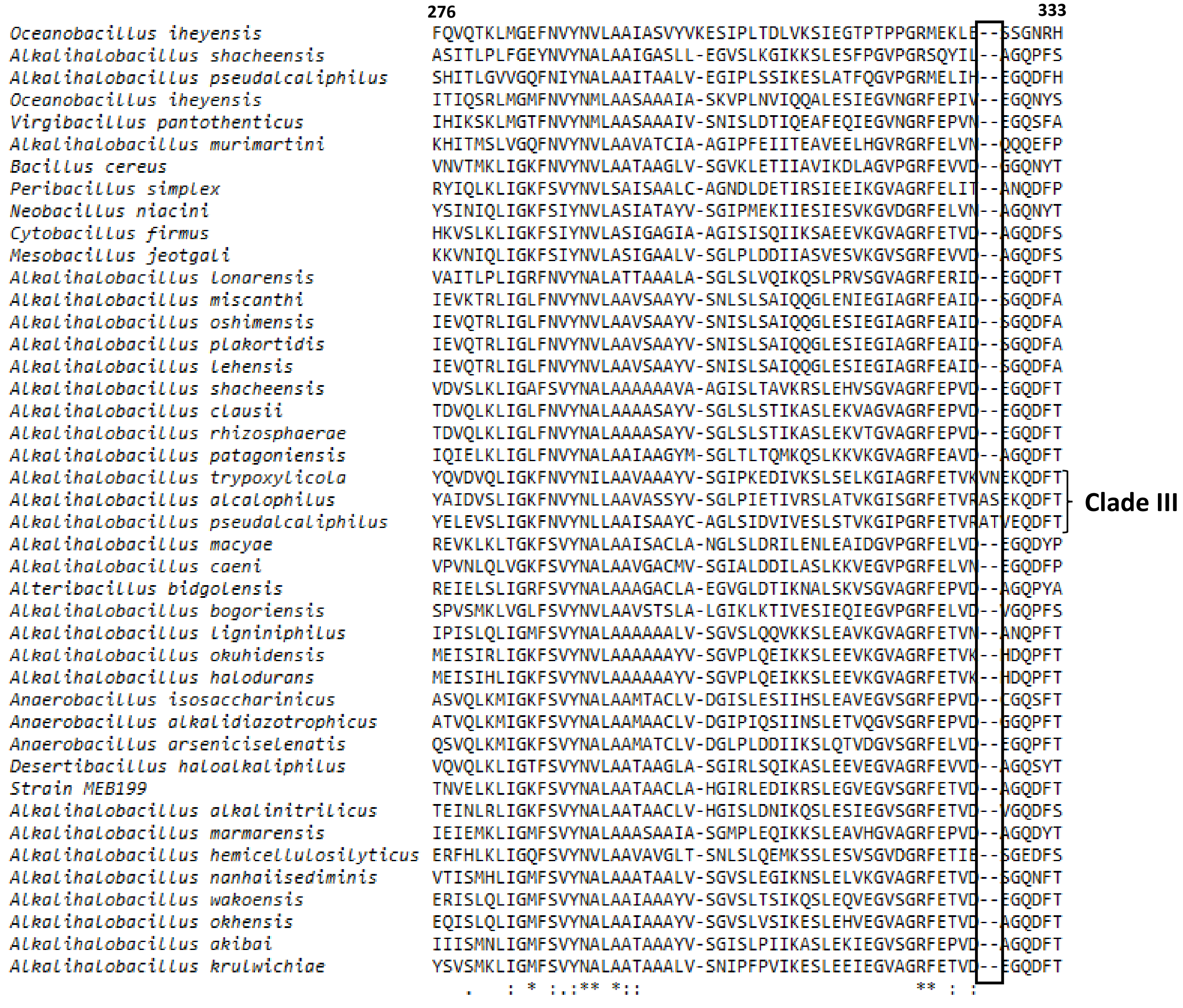

Supplementary Figure S6. A partial sequence alignment of amino acid sequence of the UDP-N-acetylmuramoyl-L-alanyl-D-glutamate-2,6-diaminopimelate ligase (murE) protein containing a amino acid insertion (boxed) that is exclusively shared by all members of the Clade III containing a homolog of this protein and absent in other members of the genus *Alkalihalobacillus*.
