# Supplementary material for: Alkalihalobacterium elongatum gen. nov. sp. nov.: An Antibiotic-Producing Bacterium Isolated From Lonar Lake and Reclassification of the Genus Alkalihalobacillus Into Seven Novel Genera
Source: Front Microbiol. 2021 Oct 11;12:722369. doi: 10.3389/fmicb.2021.722369 (PMC8543038; doi:10.3389/fmicb.2021.722369)
Supplement: Supplementary file 10 [file Image_10.PDF]

```
                                                        177                                                         237
Oceanobacillus iheyensis                                EDHIVSVEIDEVPPSMFDMLQG-SGMEQMGMNMQDAFGQFMPKKKKKR
Virgibacillus pantothenticus                            EDEIVTVEIEEQTPSMFDMLQG-SGMEHMGMNMQDALGQFMPKKKKKR
Alkalihalobacillus lonarensis                           EDREVTVEVFETKPNFMDMLPGGAGMEQMGM-MQEMFQNMMPKQKKKR ┐
Alkalihalobacillus shacheensis                          EERIVTVEVSEQNQSFMDMFQGGQGMEQMGMNMQEMLGNMMPKKKKKR │
Alkalihalobacillus clausii                              EERIVTVEVTEQNHGFMDLFQGGAGMEQMGMNMQEMLSSMMPKKKKKR │
Alkalihalobacillus rhizosphaerae                        EERTVTVEVTEQNHGFMDLFQGGAGMEQMGMNMQEMLSNMMPKKKKKR │
18 Alkalihalobacillus patagoniensis                     EDRMVTLEVSEQNQGFMDLFQGGAGMEQMGMNMQEMLGNMVPKKKKKR ├ Clade V
Alkalihalobacillus oshimensis                           EERLVTIEVSEQSQGFMDLFQGGAGMEQMGMNMQEMLGNMVPKKKKKR │
Alkalihalobacillus lehensis                             EERLVTIEVSEQSQGFMDLFQGGAGMEQMGMNMQEMLGNMVPKKKKKR │
Alkalihalobacillus plakortidis                          EERLVTIEVSEQSQGFMDLFQGGAGMEQMGMNMQEMLGNMVPKKKKKR │
Alkalihalobacillus miscanthi                            EDRVVTIEVSEQSQGFMDLFQGGAGMEQMGMNMQEMLGNMVPKKKKKR ┘
Alteribacillus bidgolensis                              EDHMVTIEVEEQAANMMDMFQG-SGMEQMGMNMQEMLGSMMPKKKKKR
Alkalihalobacillus marmarensis                          EDRIVTIEVEEQTQQFMDMFQG-AGMEQMGMNMQDMLGSMIPKKRKKR
Alkalihalobacillus trypoxylicola                        EDYMVTIEVEEQTQGFMDMFQG-AGMEQMGMNMQEMLGGMLPKKRKKR
Alkalihalobacillus alcalophilus                         EDYLVTIDVEEQQQGFMDMFQG-AGMEQMGMNMQEMLGGMMPKKRKKR
Alkalihalobacillus pseudalcaliphilus                    EDYLVNIEVEEQSQGFMDMFQG-AGMEQMGMNMQEMLGNMMPKKKKKR
Alkalihalobacillus hemicellulosilyticus                 EDHLVTIEVDEQSQNFMDMFQG-AGMEQMGMNMQEMLGGMLPKKRKKR
Alkalihalobacillus wakoensis                            EDHMVTVEMDEQSQGFMDMFQG-QGMEQMGMNMQEMLGGMLPKKKKKK
Alkalihalobacillus okhensis                             EDHMVTVEVDEQSQGFMDMFQG-QGMEQMGMNMQEMLGGMLPKKKKKR
Alkalihalobacillus nanhaiisediminis                     EDHLVTIEVDEQSQNFMDMFQG-AGMEQMGMNMQEMLGGMLPKKRKKR
Alkalihalobacillus akibai                               EDHLVTIEVDEQSQNFMDMFQG-QGMEQMGMNMQEMLGGLMPKKRKKR
Alkalihalobacillus krulwichiae                          EDHLITIEVDEQSQNFMDMFQG-AGMEQMGMNMQEMLGGLMPKKRKKR
Alkalihalobacillus ligniniphilus                        EDHYVQVEIEEQSHNLFDMFQG-AGMEQMGMNMQEMLGGMLPKKRKKR
Alkalihalobacillus okuhidensis                          EDHYVTVEVEEQTPQFFDMLQG-SGMEQMGMNMQEMLGNMMPKKRKKR
Alkalihalobacillus halodurans                           EDHYVTVEVEEQTPQFFDMLQG-SGMEQMGMNMQEMLGNMMPKKRKKR
Alkalihalobacillus macyae                               EDRMITVEVEEQNNSMMDMFQG-AGMEQMGMNMQDMLGNFMPKKKKKR
Alkalihalobacillus caeni                                EDHMVTVEVEEQSTSMFDMFQG-SGMEQMGMNMQDMLGNLVPKKKKKR
Anaerobacillus arseniciselenatis                        EDHYVTVEVEEQASGFLDMFQG-SGMEQMGMNMQEMLGNMVPKKKKKR
Anaerobacillus alkalidiazotrophicus                     EDNFVTVEVEEQTAGFLDMFQG-SGMEQMGMNMQEMLGSMIPKKKKKR
Anaerobacillus isosaccharinicus                         EDHYVTVEVEEQSGGFLDMFQG-SGMEQMGLNMQDMLGNMVPKKKKKR
Alkalihalobacillus bogoriensis                          EDWMVTIEVEEQSQNFLDMFQG-AGMEQMGINMQDMLGGMMPKRKKKR
Desertibacillus haloalkaliphilus                        EDRIVTIEVEEQSQSMFDMFQG-SGMEQMGINMQDMLGSMMPKKKKKR
Strain MEB199                                           EDHIVTIDVEEQTAGFLDMFQG-SGMEQMGINMQDMLGNMVPKKKKKR
Alkalihalobacillus alkalinitrilicus                     EDHIITIDVEEQTSGFLDMFQG-SGMEQMGINMQDMLGNMMPKKKKKR
Bacillus cereus                                         EEEIVSIEVTEQQSSMFDMLQG-TGMEQMGMNFQDALGSFMPKKTKKR
Peribacillus simplex                                    ESEMITVEVEEQAASMFDMLQG-SGMEQMGMNMQDALGSLMPKKSKKR
Mesobacillus jeotgali                                   EDELVTVEVEEQTPSMFDMLQG-SGMEQMGMNMQDALSGLVPKKRKKR
Cytobacillus firmus                                     ENEVITVEVEEQQPSMFDMLQG-SGMEQMGMNMQDALSSLMPKKRKKR
Neobacillus niacini                                     EEEIVTVEVEEQTPSMFDMLQG-SGMEQMGMNMQDALSSFMPKKRKKR
Alkalihalobacillus murimartini                          EDYYVTVEVEEQQPSMFDMLQG-SGMEQMGMNMQDALSGLMPKKKKRR
Metabacillus fastidiosus                                EDYYVVVEVEEQQPSMFDMLQG-SGMEQMGMNMQDALGSLMPKKKKKR
                                                        *.  : ::: *    ::*:: *  ***:**: :*: :  ::**: *::
```

Supplementary Figure S10. A partial sequence alignment of amino acid sequence of the ATP-dependent protease ATPase subunit (hslU) protein containing a amino acid insertion (boxed) that is exclusively shared by all members of the Clade V containing a homolog of this protein and absent in other members of the genus *Alkalihalobacillus*.
