# Supplementary material for: Alkalihalobacterium elongatum gen. nov. sp. nov.: An Antibiotic-Producing Bacterium Isolated From Lonar Lake and Reclassification of the Genus Alkalihalobacillus Into Seven Novel Genera
Source: Front Microbiol. 2021 Oct 11;12:722369. doi: 10.3389/fmicb.2021.722369 (PMC8543038; doi:10.3389/fmicb.2021.722369)
Supplement: Supplementary file 13 [file Table_3.DOCX]

| **Supplementary Table S3. antiSMASH analysis of the genus *Alkalihalobacillus*** | | | | | | | | | | | | | | | | | | | | | | | | | | | | |
| --- | --- | --- | --- | --- | --- | --- | --- | --- | --- | --- | --- | --- | --- | --- | --- | --- | --- | --- | --- | --- | --- | --- | --- | --- | --- | --- | --- | --- |
|  | **1** | **2** | **3** | **4** | **5** | **6** | **7** | **8** | **9** | **10** | **11** | **12** | **13** | 14 | **15** | **16** | **17** | **18** | 19 | 20 | 21 | 22 | 23 | 24 | 25 | 26 | 27 | 28 |
| **Siderophore** | + | + | + | - | - | - | - | - | + | - | + | - | + | + | + | + | + | + | + | + | + | + | + | + | + | + | - | - |
| **Terpene** | + | + | + | - | + | + | - | - | - | - | - | - | - | - | + | - | + | + | + | + | - | - | - | - | - | + | + | + |
| **Thailanstatin A** | + | - | - | - | - | - | - | - | - | - | - | - | - | - | - | - | - | - | - | - | - | - | - | - | - | - |  | + |
| **Lassopeptide (paeninodin)** | + | + | + | - | + | + | + | - | - | - | - | + | - | - | - | - | - | - | - | - | - | - | - | - | - | - | + | - |
| **Betalactone** | + | - | + | - | - | - | - | - | - | - | - | - | - | - | - | - | - | - | - | - | - | - | - | - | - | + | - | - |
| **Lanthipeptide-class-I (streptin)** | + | - | - | - | - | - | - | - | - | - | - | - | - | - | - | - | - | - | - | - | - | - | - | - | - | - | - | - |
| **Betalactone (fengycin)** | + | + | - | - | - | - | - | + | - | - | + | - | + | - | + | - | - | - | - | - | - | - | + | + | + | - | - | + |
| **Betalactone** | + | - | - | - | - | - | + | - | - | - | + | - | - | - | - | - | - | - | - | - | - | - | - | - | - | - | + | - |
| **T3PKS (7-deoxypactamycin)** | + | + | - | + | + | + | + | + | + | + | + | + | + | + | + | + | + | + | + | + | + | + | + | + | + | + | + | + |
| **Terpene (carotenoid)** | + | + | + | - | - | - | + | - |  |  | + | - | + |  | + | - | + | - | + | + | - | + |  |  |  | + | - | - |
| **Ectoine** | + | + | + | + | + | + | + | + | + | + | + | + | + | + | + | - | + | + | + | + | + | + | + | + | + |  | - | - |
| **LAP (RiPP-like)** | + | + | + | - | - | - | - | - | - | - | - | + | - | - | - | - | - | + | - | - | - | - | - | + | - | - | - | - |
| **Hserlactone (acinetobactin)** | - | - | - | + | - | + | - | - | - | - | - | - | - | - | - | - | - | - | - | - | - | - | - | - | - | - | - | - |
| **Ladderane** | - | - | - | - | + | - | - | - | - | - | - | - | - | - | - | - | - | + | - | - | - | - | - | - | + | - | - | - |
| **Lanthipeptide-class-iii** | - | - | - | - | + | - | - | - | - | - | - | - | + | + | - | + | + | - | + | + | - | + | - | - | - | - | - | - |
| **Thiopeptide** | - | - | - | + | - | - | - | - | - | - | - | - | - | - | - | - | - | - | - | - | - | - | - | - | - | - | - | - |
| **Petrobactin** | - | - | - | - | - | - | - | - | - | + | - | - | + | - | - | - | - | - | - | - | - | - | - | - | - | + | - | - |
| **NRPS,T1PKS** | - | - | + | - | - | - | - | - | - | - | - | - | - | + | - | - | - | + | + | + | - | + | - | - | - |  | - | - |
| **Lanthipeptide-class-I (chejuenolide A / chejuenolide B)** | - | - | - | - | - | - | - | - | - | - | - | - | - | + | - | + | - | - | - | - | + | - | - | - | - | + | - | - |
| **Furan** | - | - | - | - | - | - | - | - | - | - | - | + | - | - | - | - | - | - | - | - | - | - | - | - | - | - | - | - |
| **Cyclic-lactone-autoinducer** | - | - | - | - | - | - | - | - | - | - | - | + | - | - | - | - | - | - | - | - | - | - | - | + | + | - | - | - |
| **NRPS,T1PKS,NRPS-like** | - | - | - | - | - | - | - | - | - | - | - | + | - | - | - | - | + | - | - | + | - | - | - | - | - | - | - | - |
| **NRPS** | - | - | - | - | - | - | - | - | - | - | - | + | - | - | - | - | - | - | - |  | - | - | - | - | - | - | - | - |
| **Terpene (bacillomycin D)** | - | - | - | - | - | - | - | - | - | - | - | - | - | - | - | - | + | - | + | + | - | + | - | - | - | - | - | - |
| **Glycocin** | - | - | - | - | - | - | - | - | - | - | - | - | - | - | - | - | + | - | + | + | - | - | - | - | - | - | - | - |
| **Bacitracin** | - | - | - | - | - | - | - | - | - | - | - | - | - | + | - | - | - | - | - | - | - | - | - | - | - | - | - | - |
| **Lanthipeptide-class-iv** | - | - | - | - | - | - | - | - | - | - | - | - | - | + | - | - | - | - | - | - | - | - | - | - | - | - | - | - |
| **Lanthipeptide-class-ii (haloduracin β / haloduracin α)** | - | - | - | - | - | - | - | - | - | - | - | - | - | - | - | - | - | - | - | - | - | - | - | + | + | - | - | - |
| **Phosphonate (molybdenum cofactor)** | - | - | - | - | - | - | - | - | - | - | - | - | - | - | - | - | - | - | - | - | - | - | - | - |  | + | - | - |
| **Ranthipeptide** | - | - | - | - | - | - | - | - | - | - | - | - | - | - | - | - | - | - | - | - | - | - | - | - |  | - | - | + |
| **Sublancin RiPP:Lanthipeptide** | - | - | - | - | - | - | - | - | - | - | - | - | - | - | - | - | - | - | - | - | - | - | - | - | - | - | - | + |
| **Plipastatin** | - | - | - | - | - | - | - | - | - | - | - | - | - | - | - | - | - | - | - | - | - | - | - | - | - | - | - | + |
| **Bacillaene** | - | - | - | - | - | - | - | - | - | - | - | - | - | - | - | - | - | - | - | - | - | - | - | - | - | - | - | - |
| **Surfactin** | - | - | - | - | - | - | - | - | - | - | - | - | - | - | - | - | - | - | - | - | - | - | - | - | - | - | - | + |
| **CDPS** | - | - | - | - | - | - | - | - | - | - | - | - | - | - | - | - | - | - | - | - | - | - | - | - | - | - | - | + |
| **Subtilosin A** | - | - | - | - | - | - | - | - | - | - | - | - | - | - | - | - | - | - | - | - | - | - | - | - | - | - | - | + |
| **Bacilysin** | - | - | - | - | - | - | - | - | - | - | - | - | - | - | - | - | - | - | - | - | - | - | - | - | - | - | - | + |
| **RRE-containing** | - | - | - | - | - | - | - | - | - | - | - | - | - | - | - | - | - | - | - | - | - | + | - | - | - | - | - | + |
| **Butirosin A / butirosin B** | - | - | - | - | - | - | - | - | - | - | - | - | - | - | - | - | - | - | - | - | - | - | - | - | - | - | + | - |
| **TransAT-PKS** | - | - | - | - | - | - | - | - | - | - | - | - | - | - | - | - | - | - | - | - | - | + | - | - | - | - | - | - |

1, Strain MEB199^T^; 2, *A. alkalinitrilicus* DSM 22532^T^; 3, *A. bogoriensis* ATCC BAA-922^T^; 4, *A. alcalophilus* ATCC 27647^T^; 5, *A.*  *pseudalcaliphilus* DSM 8725^T^; 6, *A. trypoxylicola* KCTC 13244^T^; 7, *A.* *akibai* JCM 9157^T^; 8, *A. krulwichiae* AM31D^T^; 9; *A. wakoensis* JCM 9140^T^; 10, *A. okhensis* Kh10-101^T^; 11, *A. nanhaiisediminis* CGMCC 1.10116^T^, 12, *A. hemicellulosilyticus* DSM 16731^T^; 13, *A. marmarensis* DSM 21297^T^; 14, *A.* *lonarensis* 25nlg^T^; 15, *A. shacheensis* HNA-14^T^; 16, *A.* *clausii* DSM 8716^T^; 17, *A. oshimensis* DSM 18940^T^; 18, *A.* *patagoniensis* DSM 16117^T^; 19, *A.* *lehensis* DSM 19099^T^; 20, *A. plakortidis* DSM 19153; 21, *A.* *rhizosphaerae* SC-N012^T^; 22, *A. miscanthi* AK13^T^; 23, *A.* *ligniniphilus* L1^T^; 24, *A. okuhidensis* DSM 13666^T^; 25, *A.* *halodurans* DSM 497^T^; 26, *A. macyae* DSM 16346^T^; 27, *A.* *caeni* HB172195^T^, 28, *A.* *murimartini* LMG 21005^T^
