# Supplementary material for: Alkalihalobacterium elongatum gen. nov. sp. nov.: An Antibiotic-Producing Bacterium Isolated From Lonar Lake and Reclassification of the Genus Alkalihalobacillus Into Seven Novel Genera
Source: Front Microbiol. 2021 Oct 11;12:722369. doi: 10.3389/fmicb.2021.722369 (PMC8543038; doi:10.3389/fmicb.2021.722369)
Supplement: Supplementary file 8 [file Image_8.PDF]

```
                                         238                                                         295
Oceanobacillus iheyensis                 NLPVSEARKVLTQQEAQKLVDMDEVAQEATSRAEGSGIIFIDEIDKVAGKQE--NAANVS
Virgibacillus pantothenticus             KLPVAEARKVLTQQEASKLVDMEEAAQEAIQRAEQSGIVFIDEIDKVAGKEQ--QSANVS
Alkalihalobacillus lonarensis            RVTVAEARKLLIEEEAQKLIDMDDVGQEAVTRAEQIGIIFIDEIDKVVKKGE--QTADVS
18 Alkalihalobacillus shacheensis        RMSVADARKVLAEEEAQKLIDMDDVSQEAVNRTEQLGIIFIDEIDKVAGKSE--QSADVS
Alkalihalobacillus clausii               RMTVAEARGVLAEEEAQKLIDMDDVTQEAITRAEQLGIIFIDEIDKVAGKND--QGANVS
Alkalihalobacillus rhizosphaerae         RMTVAEARGVLAEEEAQKLIDMDDVTQEAITRAEQLGIIFIDEIDKVAGKND--QGANVS
Alkalihalobacillus patagoniensis         RMSVSDARGVLIEEEAQKLIDMDDVTQEAINRTEQLGMIFIDEIDKVAGKSE--QSANVS
Alkalihalobacillus oshimensis            RMSVADARIVLTEEEAQKLIDMDDVTQEAIKRAEQVGMIFIDEIDKVAGKND--QGANVS
Alkalihalobacillus lehensis              RMSVADARIVLTEEEAQKLIDMDDVTQEAIKRAEQVGMIFIDEIDKVAGKND--QGANVS
Alkalihalobacillus plakortidis           RMSVADARIVLTEEEAQKLIDMDDVTQEAIKRAEQVGMIFIDEIDKVAGKND--QGANVS
Alkalihalobacillus miscanthi             RMSVADARVVLTEEEAQKLIDMDDVTQEAIKRAEQVGMIFIDEIDKVAGKND--QGANVS
Alteribacillus bidgolensis               RLPVKDARKILTEQEAQKLIDMDEVGQLAVSKAEQLGIIFIDEIDKVTGKQQ-QSSADVS
Alkalihalobacillus marmarensis           KMPVSEARNVLAEEEAQKLIDMDEVTQLAVERAEQLGIIFIDEIDKVAGKSS-GQSADVS
Alkalihalobacillus trypoxylicola         RLPVSEARKVLTEEEAQKLIDMDEVTQEAIQKAEHLGIIFIDEIDKVAGKGN-QQSADVS
Alkalihalobacillus alcalophilus          RLPVSEARKVLTEEEAQKLIDMDEVTHDAVTKAEQLGIIFIDEIDKVAGKGN-QQSADVS
Alkalihalobacillus pseudalcaliphilus     RLPVSEARKILTEEEAQKLIDMDEVIQEALTKAEQLGIIFIDEIDKVAGKGN-QQSADVS
Alkalihalobacillus hemicellulosilyticus  RLPISDARKVLVDEEAQKLIDPDEVNQEAITKAEQLGIIFIDEIDKVASKG--QQSADVS ┐
Alkalihalobacillus wakoensis             RLPVSDARKVLIDEEAQKLIDMDEVTQEAVTRAEQLGIIFIDEIDKVASKG--QQSADVS │
Alkalihalobacillus okhensis              RLPVSDARKVLIEEEAQKLIDMDEVTQEAVLRAEQLGIIFIDEIDKVASKG--QQSADVS │
Alkalihalobacillus nanhaiisediminis      RLPVSDARKVLTDEEAQKLIDMDEVTQEAVTKAEQLGIIFIDEIDKVASKG--QQSADVS ├ Clade IV
Alkalihalobacillus akibai                RLPVSDARKVLTEEEAQKLIDMDEVTQEAVTRAEQLGIIFIDEIDKVASKG--QQSADVS │
Alkalihalobacillus krulwichiae           RLPISDARKVLIDEEAQKLIDMDEVTQEAITRAEQLGIIFIDEIDKVASKG--QQTADVS ┘
Alkalihalobacillus ligniniphilus         RLTVSEARKVLIEEEAQKLIDMDEVTQEAVSKAEQLGMVFIDEIDKVCGKSQ--QSADVS
Alkalihalobacillus okuhidensis           KLTVREARRVLTEEEAQKLIDMDEVTQEAVSKAEQLGIVFIDEIDKIAGKGQ--QSADVS
Alkalihalobacillus halodurans            KLTVREARRVLTEEEAQKLIDMDEVTQEAVSKAEQLGIVFIDEIDKIAGKGQ--QSADVS
Alkalihalobacillus macyae                KLPVSEARKVLTQDEAAKLVDMDEVAQDAVSKTEQSGIIFIDEIDKVAGKSQ--QSADVS
Alkalihalobacillus caeni                 KLPVSEARKVLTQDEASKLIDMDEVGQEAVSRAEQSGIIFIDEIDKITGKGQ--QSADVS
Anaerobacillus arseniciselenatis         KLTVREARKVLTDDEAQKLIDMDEVTQEAVLRAEQLGIIFIDEIDKVAGKSQ-QSTADVS
Anaerobacillus alkalidiazotrophicus      KLTVREARKVLTEDEAAKLIDMDEVSQEAVSRAEQLGIIFIDEIDKVAGKGN-QSTADVS
Anaerobacillus isosaccharinicus          KLTVREARKVLTEDEAQKLIDMDEVSQEAVSRAEQLGIIFIDEIDKVAGKGQ-QSTADVS
Alkalihalobacillus bogoriensis           KLTVAEARKVLTDEEAQKLIDMDEVTQDAVNRAEQLGIIFIDEIDKVAGKNQ--NSADVS
Desertibacillus haloalkaliphilus         KLPVSDARKVLTEEEAQKLIDMDEVTQEAVSKAEHLGIIFIDEIDKVAGKNQ--NSAEVS
Strain MEB199                            RLTVREARKYLTEEEAQKLIDMDEVNQEAVSKAEQLGIIFIDEIDKIAGKSQ--NNADVS
Alkalihalobacillus alkalinitrilicus      RLTVKEARKYLLEEEAQKLIDMDEVNQEAVSKAEQLGIIFIDEIDKIAGKSQ--NSADVS
Bacillus cereus                          KLSVKEARKLLSNEEAQRLIDMDEVTQEAVYRAEQLGIIFIDEIDKIAGKQS--NSVDVS
Peribacillus simplex                     KLKVSEARIVLTNEEAAKLIDMDEVTSDAVYGAEQNGIIFIDEIDKIASKQSGSSSADVS
Mesobacillus jeotgali                    KLPVREARQVLINEEAQKLIDMDEVTQEATYRAEQAGIIFIDEIDKIASKNSGGSSADVS
Cytobacillus firmus                      KLTVREARKILTNEEAQKLIDMDEVSQEAVFRAEQSGIIFIDEIDKIASKNSGGSSADVS
Neobacillus niacini                      KLTVREARKVLTNEEAARLIDMDEVTTEAVYRAEQTGIIFIDEIDKIASRNSGGSSADVS
Alkalihalobacillus murimartini           KMTVREARKVLTNEEASKLIDMDEVGQEAVQRAEESGIIFIDEIDKIAKNGGASSSADVS
Metabacillus fastidiosus                 KLTVKEARKVLTNEEATKLIDMDEVTAEAVHRAENSGIIFIDEIDKIAKKSQ-GSGADVS
                                         .: : :**  * ::** :*:* ::.   *   :*  *::*******:  .    . .:**
```

Supplementary Figure S8. A partial sequence alignment of amino acid sequence of the ATP-dependent protease ATPase subunit (hslU) protein containing a amino acid deletion (boxed) that is exclusively shared by all members of the Clade IV containing a homolog of this protein and absent in other members of the genus *Alkalihalobacillus*.
